# Hexagonal Close-Packed 2H-Cu Nanocrystals

**DOI:** 10.1021/jacs.6c09048

**Published:** 2026-07-20

**Authors:** Qingbo Wa, An Zhang, Yuhui Tian, Qinbai Yun, Changsheng Chen, Zijian Li, Li Zhai, Gemeng Liang, Biao Huang, Jun Guo, Yao Yao, Qi Yang, Wei Zhai, Huiwu Long, Hongming Xu, Peng-Fei Yin, Qipeng Lu, Jiaju Fu, Jing Xia, Minhua Shao, Wei Chen, Ye Zhu, Hua Zhang

**Affiliations:** † Department of Chemistry, 53025 City University of Hong Kong, Kowloon 999077, Hong Kong, China; ‡ Sustainable Energy and Environment Thrust, The Hong Kong University of Science and Technology (Guangzhou), Nansha, Guangzhou 511400, China; § Department of Applied Physics and Research Institute for Smart Energy, 26680 The Hong Kong Polytechnic University, Hong Kong 999077, China; ∥ School of Chemical Engineering, 1066 The University of Adelaide, South Australia 5000, Australia; ⊥ Department of Chemical and Biological Engineering & Energy Institute, 567841 The Hong Kong University of Science and Technology, Hong Kong 000000, China; # Institute of New-Energy Materials, School of Materials Science and Engineering, 12605 Tianjin University, Tianjin 300354, China; ¶ School of Materials Science and Engineering, University of Science and Technology Beijing, Beijing 100083, China; ∇ Key Laboratory of Photochemical Conversion and Optoelectronic Materials, Technical Institute of Physics and Chemistry, 74703 Chinese Academy of Sciences, Beijing 100190, China; ○ Guangzhou Key Laboratory of Electrochemical Energy Storage Technologies, Fok Ying Tung Research Institute, The Hong Kong University of Science and Technology, Guangzhou 511458, China; ⧫ Department of Chemistry, 37580 National University of Singapore, , Singapore 117549, Singapore; †† Hong Kong Branch of National Precious Metals Material Engineering Research Center (NPMM), City University of Hong Kong, Kowloon 999077, Hong Kong, China; ‡‡ Hong Kong Institute for Clean Energy, City University of Hong Kong, Kowloon 999077, Hong Kong, China; §§ Shenzhen Research Institute, City University of Hong Kong, Shenzhen 518057, China

## Abstract

Tuning the morphology and structure of Cu nanomaterials could effectively regulate their property, functions, and applications. However, it still remains challenging to directly synthesize Cu nanomaterials with an unconventional phase. Here, we report a one-pot wet-chemical synthesis of Cu nanocrystals (NCs) with a hexagonal close-packed (*hcp*, 2H type) phase, which is different from their thermodynamically stable face-centered cubic (*fcc*) phase. Compared to the conventional *fcc*-Cu NCs, the obtained 2H-Cu NCs exhibit enhanced catalytic activity and selectivity in the electrochemical carbon dioxide reduction reaction (CO_2_RR), achieving a high Faradaic efficiency (FE) of 73.1% toward multicarbon (C_2+_) products at 600 mA cm^–2^ under alkaline conditions in a flow cell. Moreover, in situ characterizations and density functional theory (DFT) calculations reveal that the 2H-Cu NCs can optimize the adsorption of the *CO intermediate, leading to a low energy barrier for the formation of C_2+_ products. This work not only demonstrates an improvement in CO_2_RR performance of Cu NCs by using the strategy of phase engineering of nanomaterials (PEN) but also opens up an avenue to explore the intrinsic properties and applications of unconventional-phase nanomaterials.

## Graphical abstract

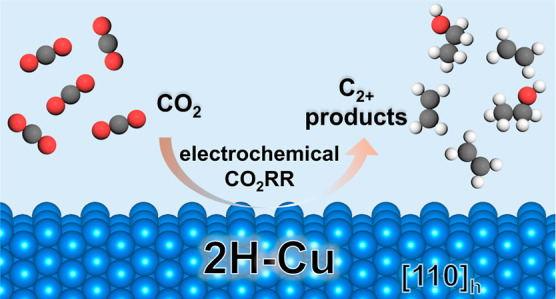

## 1 Introduction

Copper (Cu) with excellent thermal and electrical conductivities as well as high ductility is widely utilized in catalysis,^1–4^ electronics,^5,6^ photonics,^7–9^ etc. Tuning the morphology and structure of Cu has been used to modulate its properties and functions.^10–12^ For instance, the construction of a Schwarz crystal with three-dimensional minimal-interface structures in polycrystalline Cu achieved a strength approaching the theoretical value.^13^ As for Cu nanomaterials, finely tuning their structures can effectively improve their catalytic performance. For example, introducing defects, such as grain boundaries and twin boundaries, into the perfect crystal structure of Cu nanomaterials has significantly improved their catalytic activity and selectivity in the electrochemical carbon dioxide reduction reaction (CO_2_RR).^14,15^ However, these achievements have only been obtained in the face-centered cubic (*fcc*) phase, i.e., the thermodynamically stable phase, of Cu. The change of the crystal phase of Cu nanomaterials still remains challenging.

Recently, phase engineering of nanomaterials (PEN) has become an effective strategy to change the intrinsic physicochemical properties and enhance application performances through regulating the long-range atomic arrangements, i.e., crystal phases, of nanomaterials.^16–22^ In particular, the unique surface coordination environments and electronic structures of unconventional-phase nanomaterials would endow them with enhanced catalytic properties compared to their counterparts with conventional thermodynamically stable phases.^23^ Normally, Cu crystallizes in its thermodynamically stable *fcc* phase. Although the epitaxial growth method has been used to prepare Cu nanomaterials with an unconventional 4H phase, and 4H/*fcc* and *fcc*-2H-*fcc* heterophases on the corresponding Au templates,^24–26^ the direct synthesis of unconventional-phase Cu nanocrystals (NCs) is still challenging due to their thermodynamically unstable nature. Therefore, it is highly desirable to directly synthesize unconventional-phase Cu with high phase purity and then explore their intrinsic phase-dependent properties and applications.

In this work, we report a one-pot wet-chemical synthesis of unconventional hexagonal close-packed (*hcp*, 2H-type) Cu NCs, referred to as 2H-Cu NCs, with high phase purity. As a proof-of-concept application, the obtained 2H-Cu NCs are used as an electrocatalyst for a highly efficient CO_2_RR with superior catalytic activity and selectivity toward multicarbon (C_2+_) products, outperforming the most widely studied thermodynamically stable *fcc*-Cu NCs. Impressively, the 2H-Cu NCs achieve a high Faradaic efficiency (FE) of 72.4% at −1.30 V versus the reversible hydrogen electrode (vs RHE) toward C_2+_ products under neutral conditions in an H-type cell, as well as an excellent FE of 73.1% for C_2+_ products at 600 mA cm^–2^ under alkaline conditions in a flow cell. In situ characterization and theoretical calculation results reveal that the unconventional 2H-Cu significantly lowers the energy barrier for both *CO and *CHO formation compared to the *fcc*-Cu, which facilitates an energy-favorable asymmetric *CO–*CHO coupling pathway for the generation of C_2+_ products in the CO_2_RR.

## 2 Results and Discussion

### 2.1 Synthesis and Characterization of 2H-Cu NCs

The unconventional 2H-Cu NCs were synthesized via a one-pot wet-chemical method (see Supporting Information for details). Figure 1a illustrates the crystal structures of unconventional 2H-Cu and conventional *fcc*-Cu with typical “AB” and “ABC” stacking sequences, respectively. The transmission electron microscopy (TEM) image of 2H-Cu NCs shows their morphology (Figure 1b) with a size of 63.4 ± 7.8 nm (Figure S1). The spherical aberration-corrected high-angle annular dark-field scanning TEM (HAADF-STEM) image of a representative 2H-Cu NC features a characteristic atomic arrangement of the 2H phase, exhibiting an “AB” stacking sequence along the [002]_h_ close-packed direction viewed from the [110]_h_-zone axis (Figure 1c), which is consistent with the structural model of the 2H phase shown in Figure 1a. The selected-area electron diffraction (SAED) pattern (inset of Figure 1b) and the corresponding fast Fourier transform (FFT) pattern (inset of Figures 1c and S2) further confirm the formation of the unconventional 2H phase. Moreover, the X-ray diffraction (XRD) pattern of 2H-Cu NCs matches well with the simulated pattern, and no additional diffraction peak can be observed (Figure 1d), indicating their high phase purity. Meanwhile, the Rietveld refinement analysis in the XRD pattern (Figure S3) suggests that 2H-Cu belongs to a hexagonal structure, which crystallizes in the *P*6_3_/*mmc* (194) space group with the lattice parameters of *a* = *b* = 2.57 Å and *c* = 4.21 Å (Table S1).

**Figure 1:**
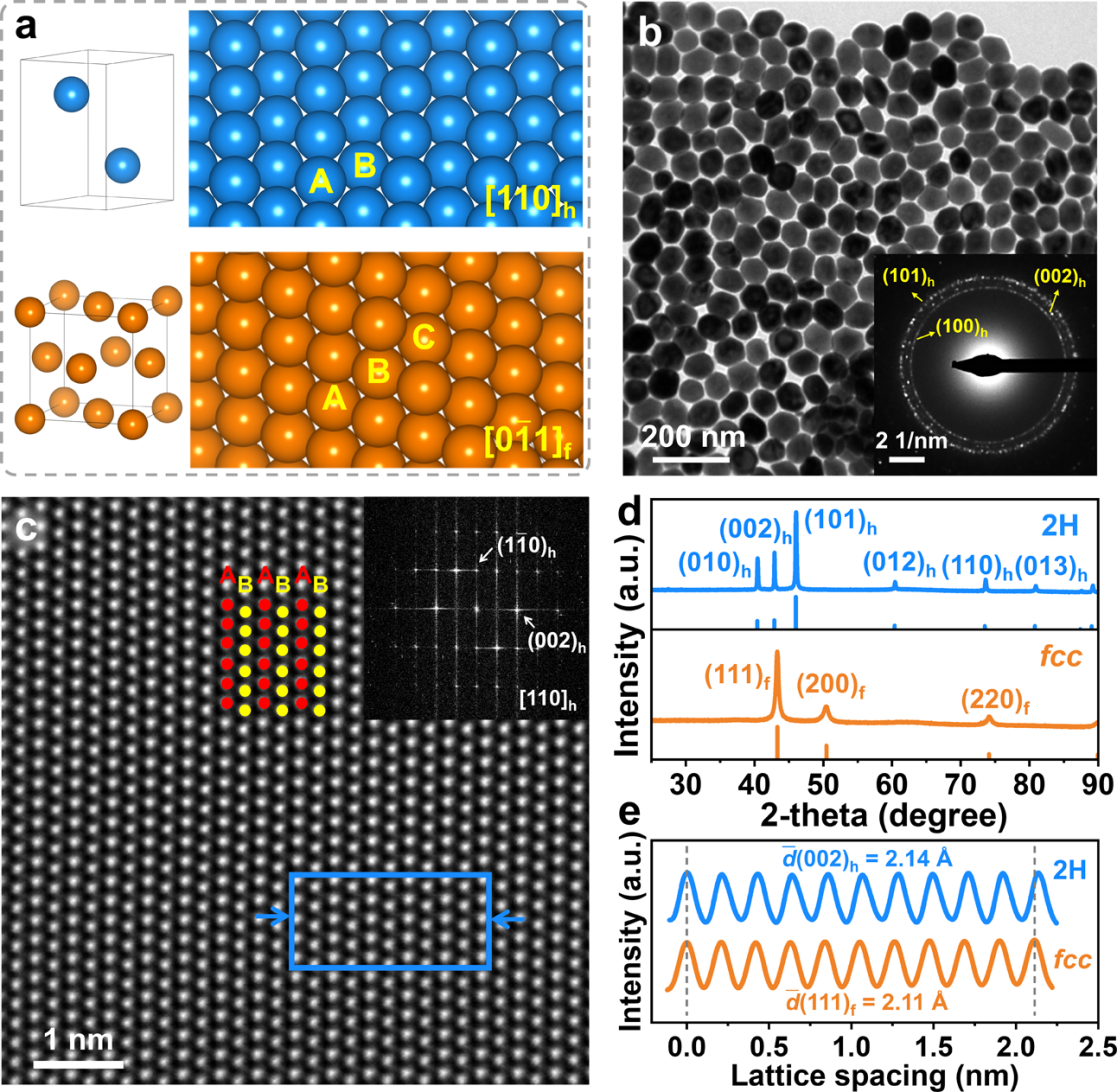
(a) Crystal structures and stacking models (viewed along [110]_h_/[01̅1]_f_ zone axes) of 2H- (upper panel) and *fcc*-Cu (lower panel). (b) TEM image and (c) spherical aberration-corrected HAADF-STEM image of 2H-Cu NCs. Inset in (b): the corresponding SAED pattern of 2H-Cu NCs. Inset in (c): the corresponding FFT pattern of 2H-Cu NC. (d) XRD patterns of 2H-Cu and *fcc*-Cu NCs. (e) The integrated pixel intensities along the arrow directions of the selected areas in (c) and Figure S4c, respectively.

In order to investigate the effect of the crystal phase on the intrinsic properties of Cu NCs, the conventional *fcc*-Cu NCs with a similar morphology and size of 71.1 ± 7.4 nm were also synthesized and characterized (Figures S4 and S5). The *fcc*-Cu NCs exhibit a typical “ABC” stacking sequence along the [111]_f_ close-packed direction as shown in the HAADF-STEM image (Figure S4c), which is consistent with the structural model of *fcc*-Cu (Figure 1a). To further compare the structural differences between 2H-Cu and *fcc*-Cu NCs, the interplanar spacings between their close-packed planes, i.e., 2H (002)_h_ and *fcc* (111)_f_, were investigated (Figure 1e). Based on the integrated pixel intensities of 2H (002)_h_ and *fcc* (111)_f_ lattices from the selected areas in the corresponding HAADF-STEM images, the average interlayer spacing of 2H (002)_h_ planes is 2.14 Å, slightly larger (~1.4%) than that of *fcc* (111)_f_ planes (2.11 Å), aligning well with the peaks of these two planes observed in the XRD patterns of 2H-Cu and *fcc*-Cu NCs (Figure 1d).

X-ray photoelectron spectroscopy (XPS) was used to investigate the valence states of 2H-Cu and *fcc*-Cu NCs (Figure 2a). The Cu 2p peaks located at 952.2 eV (Cu 2p_1/2_) and 932.5 eV (Cu 2p_3/2_) can be observed in both Cu NCs, indicating their metallic state.^27,28^ Moreover, the electronic structures and coordination environments of 2H-Cu and *fcc*-Cu NCs were further analyzed by X-ray absorption spectroscopy. As shown in the normalized X-ray absorption near-edge structure (XANES) spectra (Figure 2b), the absorption edge positions of the 2H-Cu and *fcc*-Cu NCs are close to that of Cu foil, confirming their metallic state, which is consistent with the XPS results (Figure 2a). Meanwhile, the Fourier-transformed extended X-ray absorption fine structure (EXAFS) spectra of 2H-Cu and *fcc*-Cu NCs show similar Cu–Cu scattering paths to those of Cu foil (Figure 2c). As shown in Figure S6 and Table S2, the fitting results indicate that 2H-Cu NCs feature a slightly larger Cu–Cu distance compared to the *fcc*-Cu NCs and Cu foil, which is consistent with the observed larger lattice parameter of 2H-Cu in HAADF-STEM data (Figures 1c,e, S4c) and XRD results (Figure 1d). In addition, the intensity maxima of a wavelet transform (WT) of the Cu K-edge EXAFS spectrum of 2H-Cu NCs (Figure 2d) are very close to those of *fcc*-Cu NCs (Figure 2e) and Cu foil (Figure 2f), confirming their metallic state.

**Figure 2:**
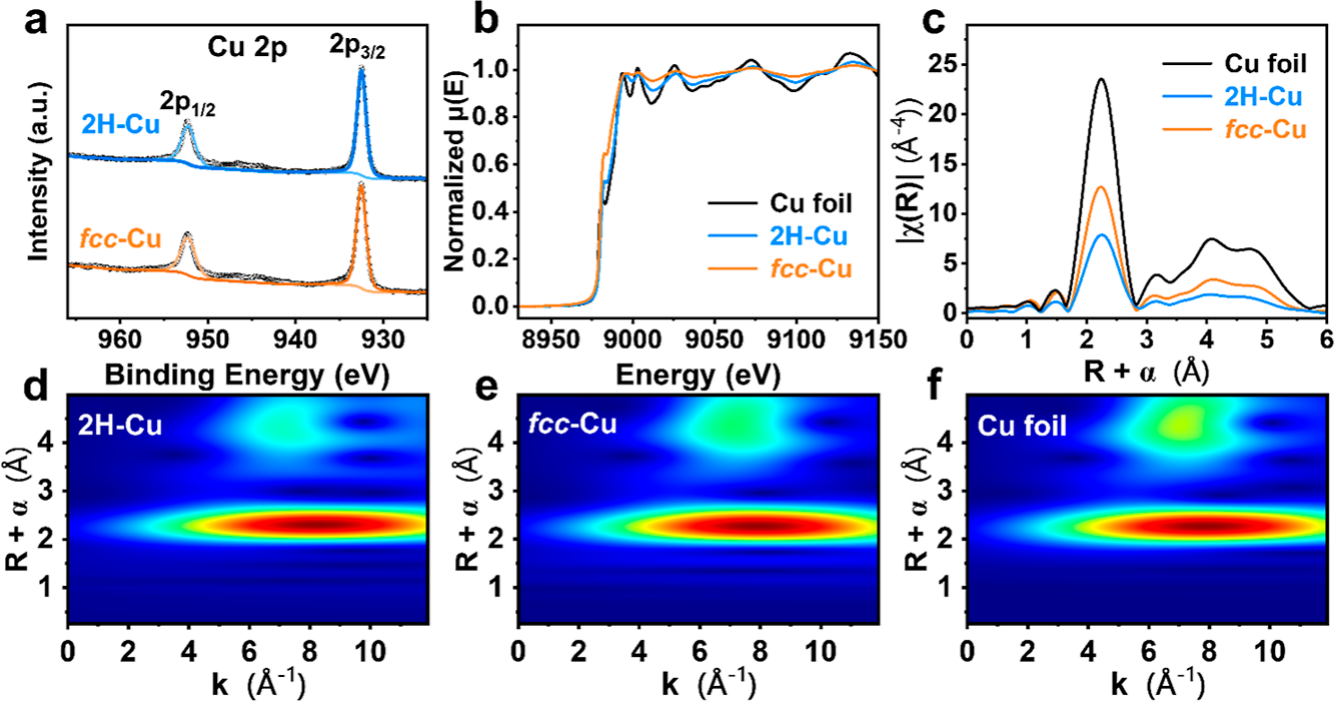
(a) Cu 2p XPS spectra of 2H-Cu and *fcc*-Cu NCs. (b) XANES spectra and (c) Fourier-transformed EXAFS spectra of 2H-Cu NCs, *fcc*-Cu NCs, and Cu foil. (d–f) Wavelet transform of Cu K-edge EXAFS spectra for 2H-Cu NCs (d), *fcc*-Cu NCs (e), and Cu foil (f).

Moreover, the thermal stability of 2H-Cu NCs was investigated by annealing them at different temperatures in an Ar atmosphere. As shown in Figure S7, the 2H-Cu NCs exhibit a 2H-to-*fcc* phase transition at the annealing temperature of ~250 °C, indicating their good thermal stability compared to 2H-Au nanomaterials.^29^ A mixture of 2H and *fcc* phases can be observed when the annealing temperature increases from 250 to 275 °C. When the annealing temperature reaches ~300 °C, the 2H phase completely transforms to the thermodynamically stable *fcc* phase.

### 2.2 Electrocatalytic CO_2_RR Performance of 2H-Cu NCs

As is known, Cu has been used as an effective electrocatalyst to reduce CO_2_ into high-value C_2+_ products (e.g., ethylene, ethanol, and propanol).^30–35^ However, the selectivity to a specific C_2+_ product is still low, limiting the practical application of the CO_2_RR. As a proof-of-concept application, the electrocatalytic CO_2_RR performance of as-synthesized 2H-Cu NCs was evaluated. For comparison, the CO_2_RR electrocatalyzed by the conventional *fcc*-Cu NCs was also measured. The obtained gaseous products during the CO_2_RR were detected by online gas chromatography, and the obtained liquid products were analyzed by ^1^H nuclear magnetic resonance spectroscopy. As shown in Figure S8, the linear sweep voltammetry curves indicate that the 2H-Cu NCs deliver a higher current density compared to the *fcc*-Cu NCs, suggesting a higher CO_2_RR activity. As shown in Figure 3a,b, similar types of CO_2_RR products, e.g., gaseous products of carbon monoxide (CO), methane (CH_4_), and ethylene (C_2_H_4_), as well as the liquid products of formate (HCOO^–^), ethanol (C_2_H_5_OH), acetate (CH_3_COO^–^), and n-propanol (*n*-C_3_H_7_OH), have been obtained on 2H-Cu and *fcc*-Cu NCs. Specifically, 2H-Cu NCs exhibit enhanced selectivity toward the C_2_H_4_ and C_2+_ products compared to the *fcc*-Cu NCs, achieving the maximum FE_C2H4_ of 50.3% and FE_C2+_ of 72.4% at −1.30 V (vs RHE) in an H-type cell (Figure 3c), placing our 2H-Cu NCs among the best Cu-based CO_2_RR catalysts (Table S3). Compared to the *fcc*-Cu NCs, the 2H-Cu NCs achieve higher partial current densities for C_2_H_4_ and C_2+_ products (Figure S9). Notably, the 2H-Cu NCs also exhibit an apparently higher FE_CO_ than the *fcc*-Cu NCs under low overpotentials (Figure 3a,b). As the overpotential increases, the FE_CO_ rapidly decreases with the increase of FE_C2H4_, implying that CO might be a key intermediate for producing C_2_H_4_. In addition, the higher FE_CH4_ on 2H-Cu NCs compared to *fcc*-Cu NCs observed under higher overpotentials suggests that the 2H-Cu NCs may exhibit a higher activity for CO protonation, which would also lower the energy barrier for C–C coupling based on the previous report.^36^ Importantly, 2H-Cu NCs were found to effectively suppress the competitive hydrogen evolution reaction (HER) as indicated by their decreased partial current density of the H_2_ product compared to *fcc*-Cu NCs (Figure S10). Moreover, the 2H-Cu NCs exhibit a greater electrochemically active surface area (ECSA) of 8.52 × 10^3^ cm^2^ g^–1^ compared to the *fcc*-Cu (8.33 × 10^3^ cm^2^ g^–1^, Figure S11). In addition, 2H-Cu delivers higher ECSA-normalized partial current densities for both C_2_H_4_ and total C_2+_ products, confirming its higher intrinsic catalytic activity.

**Figure 3:**
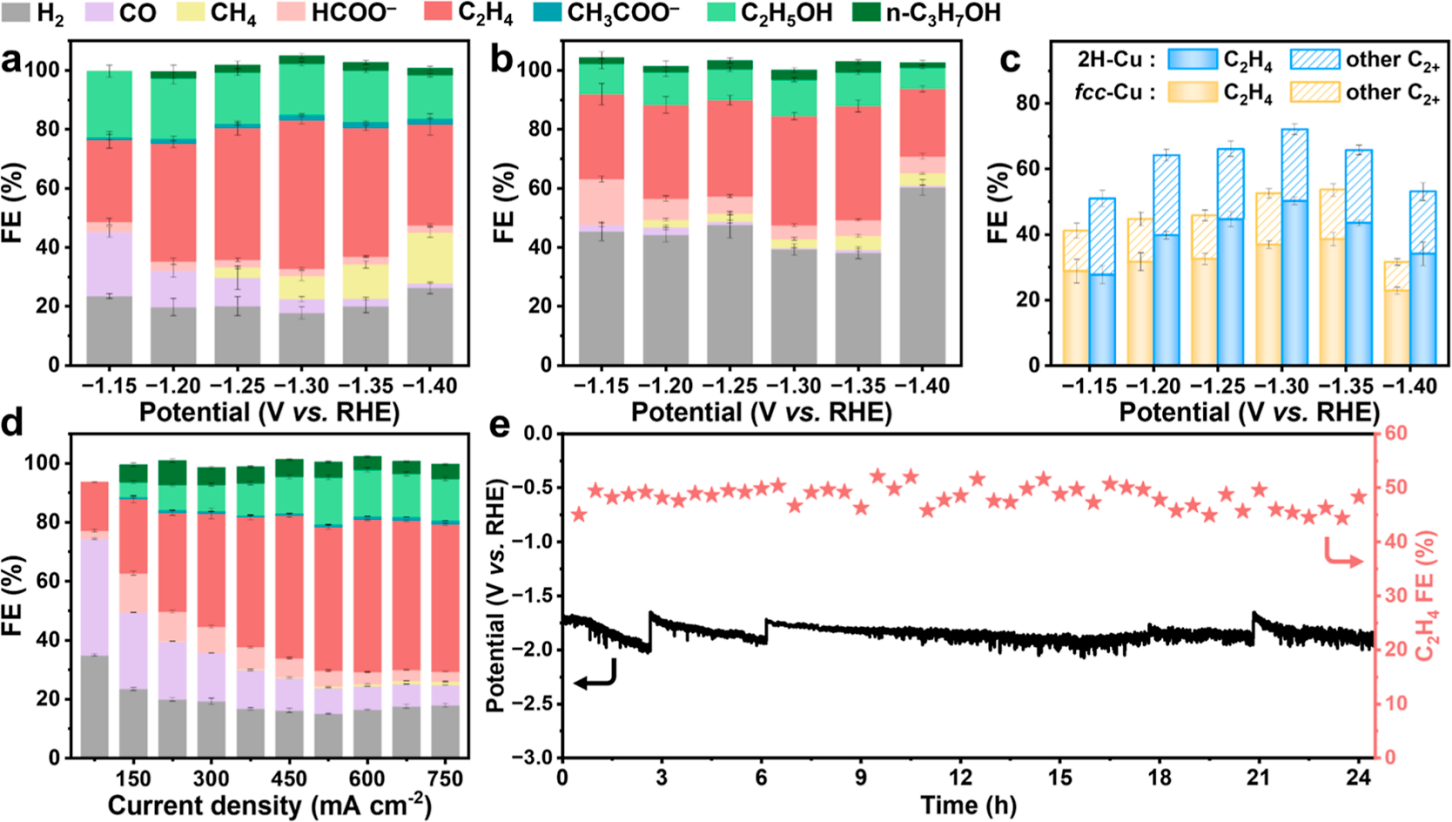
(a,b) Potential-dependent product distributions of the CO_2_RR on 2H-Cu (a) and *fcc*-Cu (b) NCs in 0.1 M KHCO_3_ electrolytes in an H-type cell. (c) FEs for C_2_H_4_ and C_2+_ products on 2H-Cu and *fcc*-Cu NCs under different potentials in 0.1 M KHCO_3_ electrolytes in an H-type cell. (d) Current-density-dependent product distributions of the CO_2_RR on 2H-Cu NCs in 1 M KOH electrolytes in a flow cell. (e) The long-term stability test of 2H-Cu NCs in a flow cell at a current density of 500 mA cm^–2^.

Moreover, the CO_2_RR performance of the 2H-Cu NCs under industrial-level current densities was also investigated in a flow cell. Notably, the catalyst exhibits the highest FE_C2H4_ of 51.7% and FE_C2+_ of 73.0% at 600 mA cm^–2^ (Figure 3d) as well as a high partial current density toward C_2+_ products (*J*_C2+_) of 526 mA cm^–2^ at −2.41 V vs RHE (Figure S12). In addition, the chronopotentiometry test of 2H-Cu NCs was also evaluated at 500 mA cm^–2^, showing that both the overpotential and FE_C2H4_ are well maintained over 24 h of electrocatalysis (Figures 3e and S13). To the best of our knowledge, these results place the 2H-Cu NCs among the best CO_2_RR catalysts toward C_2+_ products (Table S4), indicating their great potential for practical applications.

### 2.3 In Situ Characterization and Theoretical Calculation

In order to understand the phase-dependent electrocatalytic activities and selectivities of 2H- and *fcc*-Cu, in situ attenuated total reflectance Fourier transform infrared (ATR-FTIR) spectroscopy was used to monitor the key adsorbed intermediates during the electrocatalytic CO_2_RR on 2H- and *fcc*-Cu NCs (Figure 4a,b). As shown in Figure 4a, there are two obvious adsorption peaks at ~2058 and 1806 cm^–1^, which can be assigned to the C=O stretching in atop-adsorbed *CO (*CO_atop_) and bridge-adsorbed *CO (*CO_bridge_), respectively,^37–39^ on the surface of 2H-Cu NCs. In contrast, the *fcc-*Cu NCs mainly show the *CO_atop_ peak together with a weak signal of *CO_bridge_, indicating the different *CO binding configurations on 2H- and *fcc*-Cu. In particular, the *CO_atop_ adsorption peak on 2H-Cu NCs is stronger than that on *fcc*-Cu NCs, demonstrating the higher *CO coverage on 2H-Cu. Meanwhile, the key intermediates of *COOH (∼1450 and 1255 cm^–1^)^40,41^ during the *CO generation can be identified on both 2H- and *fcc*-Cu NCs. Moreover, based on the previous reports,^42–45^ the only observation of the *CHO intermediate (∼1675 cm^–1^) on 2H-Cu NCs indicates that *CHO is more likely to form on the surface of 2H-Cu. This can be attributed to the lower energy barrier of *CO_bridge_ protonation than that of *CO_atop_ on the Cu surface,^46–48^ which is also consistent with the stronger signal of the *CO_bridge_ intermediate on 2H-Cu NCs. Consequently, the high *CO coverage and energetically favorable *CHO formation on 2H-Cu NCs facilitate the subsequent asymmetric C–C coupling to generate the *COCHO intermediate (∼1562 cm^–1^),^42,47,49^ thereby enhancing the selectivity of C_2+_ products.^40,50^

**Figure 4:**
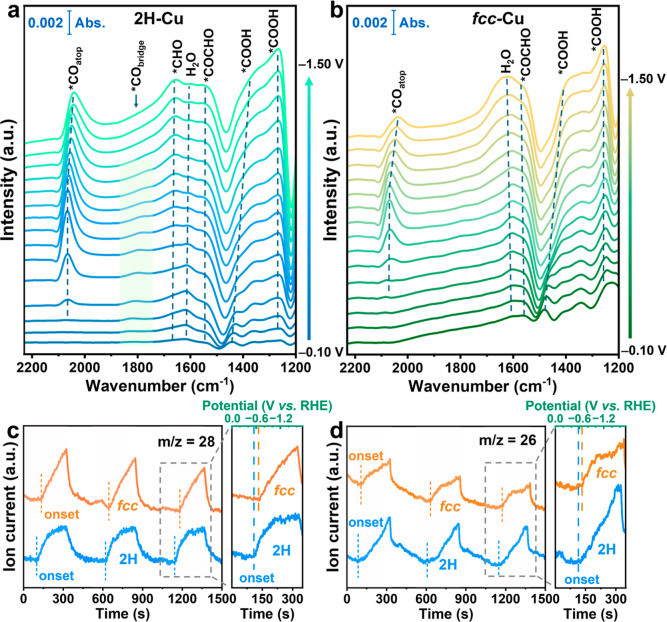
(a,b) In situ ATR-FTIR spectra of the CO_2_RR on 2H-Cu (a) and *fcc*-Cu (b) NCs. (c,d) In situ DEMS characteristic patterns of CO (c) and C_2_H_4_ (d) on 2H-Cu (blue curves) and *fcc*-Cu (orange curves) NCs.

To better track the formation of different products, in situ differential electrochemical mass spectrometry (DEMS) was used to probe the electrocatalytic CO_2_RR on 2H- and *fcc*-Cu NCs, with the applied cathodic potentials from 0 to −1.60 V (vs RHE). The characteristic mass-to-charge signals for the fragments are associated with C_2_H_4_ (C_2_H_2_^+^, *m*/*z* = 26) and CO (*m*/*z* = 28).^51–55^ As shown in Figure 4c, the typical signal assigned to the CO species appears earlier on 2H-Cu NCs compared to *fcc*-Cu NCs, suggesting that the formation of CO would encounter a lower barrier on the 2H-Cu NCs (consistent with the higher FE_CO_ at low overpotentials shown in Figure 3a), which can serve as essential source/intermediate for the following C–C coupling to generate C_2+_ products. As a result, C_2_H_4_ is generated more readily on 2H-Cu NCs, leading to a lower overpotential in the DEMS test compared to *fcc*-Cu NCs (Figure 4d). These results further confirm that the unconventional 2H phase could promote the generation of C_2+_ products on Cu during the electrocatalytic CO_2_RR.

Furthermore, density functional theory (DFT) calculations were performed to gain deep insights into the effect of the crystal phase on the electrocatalytic CO_2_RR process of Cu. In particular, the distinct facets of 2H- and *fcc*-Cu, i.e., 2H (110)_h_ and (101)_h_, and *fcc* (100)_f_ and (111)_f_, which were observed by TEM (Figures 1c, S2, and S5), were selected as models for calculations. As shown in the projected partial density of states (PDOSs) spectra (Figure 5a), the Cu-3d orbitals of 2H-Cu (d-band centers of −2.29 eV and −2.28 eV for (110)_h_ and (101)_h_, respectively) are upshifted closer to the Fermi level compared to the *fcc*-Cu (d-band centers of −2.32 eV and −2.34 eV for (111)_f_ and (100)_f_, respectively). This could result in stronger interactions between the 2H-Cu surface and the adsorbates in the CO_2_RR.^43,56^ Meanwhile, the energy barriers of key intermediates during the electrocatalytic CO_2_RR were also investigated. First, the energy barrier for the conversion of CO_2_ to *CO was compared on (110)_h_ and (101)_h_ of 2H-Cu and (111)_f_ and (100)_f_ of *fcc*-Cu (Figures 5b, S14). As is known, the formation of *COOH from CO_2_ should be the key intermediate in converting CO_2_ to *CO.^57–60^ As shown in Figure 5b, the (110)_h_ and (101)_h_ facets of 2H-Cu possess low energy barriers (0.39 and 0.47 eV, respectively) for the *COOH formation, much lower than those of the *fcc* (111)_f_ (0.66 eV) and (100)_f_ (0.61 eV). These results suggest that the unconventional 2H-Cu could enhance the CO generation, consistent with our experimental CO_2_RR results (Figure 3a,c) and in situ FTIR results (Figure 4a).

**Figure 5:**
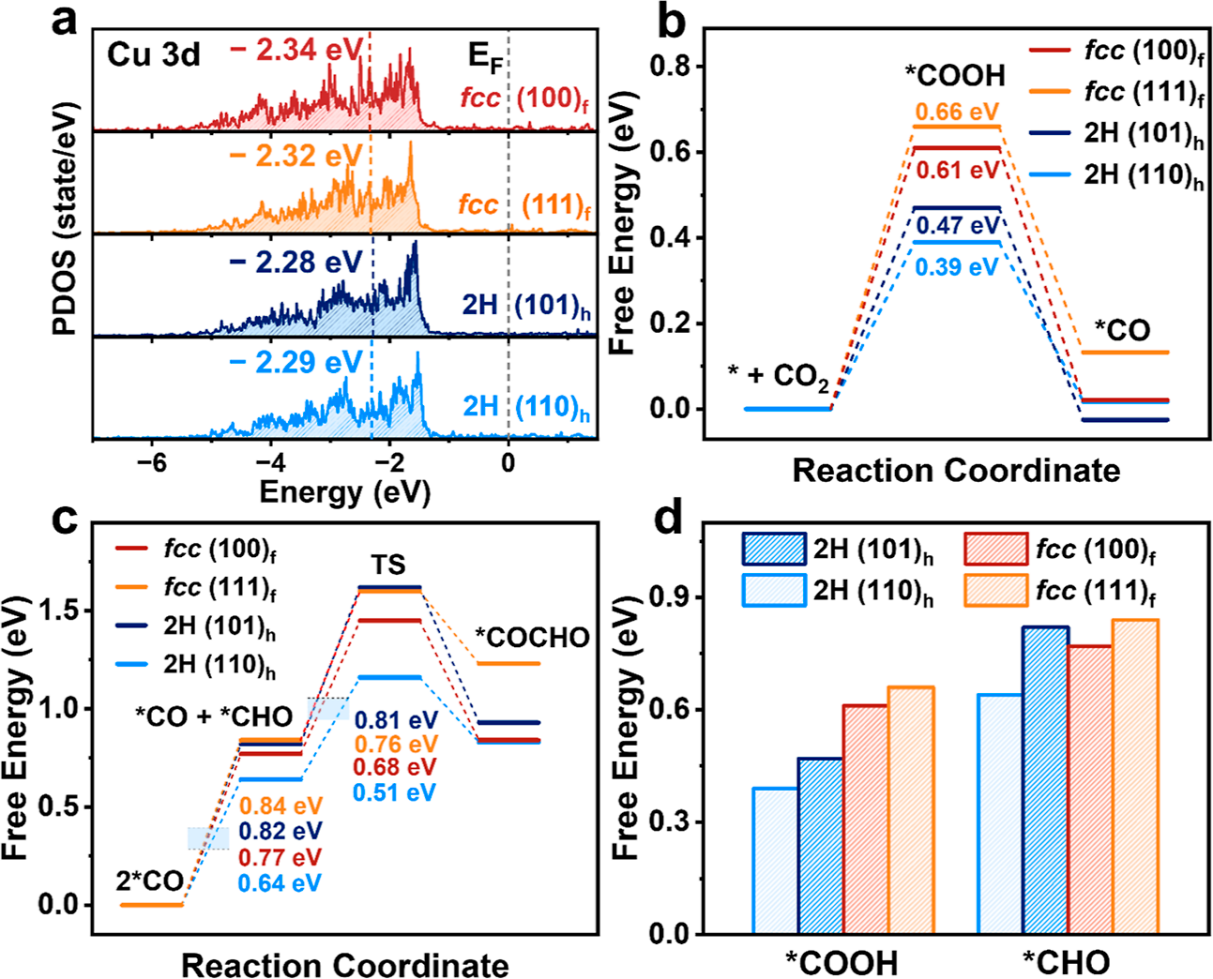
(a) PDOSs of *fcc*-Cu (100)_f_, *fcc*-Cu (111)_f_, 2H-Cu (101)_h_, and 2H-Cu (110)_h_ facets. (b) Free energy diagrams of *fcc*-Cu (100)_f_, *fcc*-Cu (111)_f_, 2H-Cu (101)_h_, and 2H-Cu (110)_h_ facets for CO_2_ reduction via the formation of *COOH to produce *CO. (c) Free energy diagrams of *fcc*-Cu (100)_f_, *fcc*-Cu (111)_f_, 2H-Cu (101)_h_, and 2H-Cu (110)_h_ facets for asymmetric coupling of *CO and *CHO to generate the *COCHO intermediate. (d) Summary of free energy barriers of *COOH and *CHO intermediates on *fcc*-Cu (100)_f_, *fcc*-Cu (111)_f_, 2H-Cu (101)_h_, and 2H-Cu (110)_h_ facets.

In addition, the energy barrier of C–C coupling was calculated to further illustrate the reaction pathways for C_2+_ products (i.e., C_2_H_4_ and C_2_H_5_OH). Previous studies have found that the direct coupling of two *CO usually suffers from a high energy barrier.^61–64^ Therefore, the pathway for hydrogenation of *CO to form the *CHO intermediate^31,42,65^ was selected on (110)_h_ and (101)_h_ of 2H-Cu and (111)_f_ and (100)_f_ of *fcc*-Cu (Figure S15). As shown in Figure 5c, the formation of *CHO is the rate-determining step (RDS), followed by the asymmetric coupling of *CO and *CHO to generate the *COCHO intermediate.^42^ Compared to *fcc*-Cu, 2H-Cu delivers an obviously decreased energy barrier for the formation of *CHO, as well as the further asymmetric coupling of *CO and *CHO, which is consistent with the in situ FTIR results (Figure 4a,b). These calculation results certify that the C_2+_ reaction pathway is more energetically favored on the unconventional 2H-Cu. Overall, the lower energy barriers of these key intermediates (*COOH and *CHO) on the 2H-Cu surface (Figure 5d) lead to its higher efficiency and selectivity toward the C_2+_ products compared to *fcc*-Cu.

## 3 Conclusions

In summary, we have successfully synthesized unconventional-phase 2H-Cu NCs with high phase purity via a one-pot wet-chemical method. When used as catalysts for the electrochemical CO_2_RR, the obtained 2H-Cu NCs exhibit higher catalytic activity and enhanced selectivity toward high-value C_2+_ products (72.4% at −1.30 V vs RHE), especially for the C_2_H_4_ product (50.3% at −1.30 V vs RHE) under near-neutral electrolytes, compared to the conventional thermodynamically stable *fcc*-Cu NCs. Notably, the 2H-Cu NCs also achieve a competitive Faradaic efficiency (73.1% at 600 mA cm^–2^) toward C_2+_ products in the flow cell, placing them among the best CO_2_RR catalysts for C_2+_ products. The in situ ATR-FTIR and in situ DEMS results show that 2H-Cu exhibits a lower onset potential for CO formation compared to *fcc*-Cu, leading to high *CO coverage and promoting the formation of *CHO intermediates. Theoretical calculations further demonstrate that the unconventional 2H-Cu significantly lowers the energy barrier for both *CO and *CHO formation compared to *fcc*-Cu, which facilitates an energy-favorable asymmetric *CO–*CHO coupling pathway for the generation of C_2+_ products in the CO_2_RR. This work not only opens up an avenue for the development of unconventional-phase metal nanomaterials but also shows great potential to boost the electrocatalytic performance via the PEN strategy.

## Supplementary Material



## Notes

Chemicals, detailed experimental methods, characterizations, electrochemical measurements, computational details, additional figures, and tables (PDF)
