# Supplementary material for: Hexagonal Close-Packed 2H-Cu Nanocrystals
Source: J Am Chem Soc. 2026 Jul 20;148(29):31413–21. doi: 10.1021/jacs.6c09048 (PMC13426263; doi:10.1021/jacs.6c09048)
Supplement: Supplementary file 1 [file ja6c09048_si_001.pdf]

## Supporting Information

# Hexagonal Close-Packed 2H-Cu Nanocrystals

Qingbo Wa[1,#], An Zhang[1,#], Yuhui Tian[1,#], Qinbai Yun[1,2,*], Changsheng Chen[3], Zijian Li[1], Li Zhai[1], Gemeng Liang[4], Biao Huang[1], Jun Guo[1], Yao Yao[1], Qi Yang[1], Wei Zhai[1], Huiwu Long[1], Hongming Xu[1,5], Peng-Fei Yin[1,6], Qipeng Lu[7], Jiaju Fu[1], Jing Xia[8], Minhua Shao[5,9], Wei Chen[10], Ye Zhu[3], Hua Zhang[1,11,12,13*]

[1]Department of Chemistry, City University of Hong Kong, Kowloon, Hong Kong, China.

[2]Sustainable Energy and Environment Thrust, The Hong Kong University of Science and Technology (Guangzhou), Nansha, Guangzhou 511400, China.

[3]Department of Applied Physics and Research Institute for Smart Energy, The Hong Kong Polytechnic University, Hong Kong, China.

[4]School of Chemical Engineering, The University of Adelaide, SA, 5000, Australia.

[5]Department of Chemical and Biological Engineering & Energy Institute, The Hong Kong University of Science and Technology, Hong Kong, China.

[6]Institute of New-Energy Materials, School of Materials Science and Engineering, Tianjin University, Tianjin, 300354, China

[7]School of Materials Science and Engineering, University of Science and Technology Beijing, Beijing, 100083, China.

[8]Key Laboratory of Photochemical Conversion and Optoelectronic Materials, Technical Institute of Physics and Chemistry, Chinese Academy of Sciences, Beijing, 100190, China.

[9]Guangzhou Key Laboratory of Electrochemical Energy Storage Technologies, Fok Ying Tung Research Institute, The Hong Kong University of Science and Technology, Guangzhou 511458, China.

[10]Department of Chemistry, National University of Singapore, 117549, Singapore.

[11]Hong Kong Branch of National Precious Metals Material Engineering Research Center (NPMM), City University of Hong Kong, Kowloon, Hong Kong, China.
[12]Hong Kong Institute for Clean Energy, City University of Hong Kong, Kowloon, Hong Kong, China.
[13]Shenzhen Research Institute, City University of Hong Kong, Shenzhen 518057, China.

[#]These authors contributed equally to this work.

[*]Corresponding author.

Email address: hua.zhang@cityu.edu.hk; qinbaiyun@hkust-gz.edu.cn

## Methods

### Chemicals and reagents

Copper(II) acetylacetonate ($Cu(acac)_2$, 97%), trioctylphosphine oxide (TOPO, ReagentPlus, 99%), oleylamine (OAm, ≥98%), octylamine (OctA, 99%) were purchased from Sigma-Alarich. Hydrobromic acid (HBr, 48%), L-Ascorbic acid (AA, 99%), hexane (99%), ethanol (absolute, ≥ 99.9%), isopropanol (99%), $D_2O$ (99.9 atom% D) and dimethylsulfoxide (DMSO, ≥ 99.9%) were purchased from Aladdin. Carbon dioxide (99.999%) gas cylinders were purchased from Specialty Gas Engineering Co. Ltd. All the chemicals and reagents were used as received without any further purification. The Milli-Q water (resistivity of 18.2 MΩ·cm; Milli-Q System, Millipore) was used in our experiment.

### Characterizations

X-ray diffraction (XRD) was carried out on a Rigakur SmartLab equipped with a Cu $K_{\alpha 1}$ radiation ($\lambda$ =1.54 Å). Transmission electron microscopic (TEM) images were collected by a JEM-2100F microscope (JEOL, Japan) under an acceleration voltage of 200 KV. X-ray photoelectron spectroscopy (XPS) measurements were conducted on the ESCALAB 250Xi (Thermo Fisher Scientific) instrument. The C1s with a binding energy of 284.8 eV was used as the reference. The inductively coupled plasma optical emission spectrometry (ICP-OES) analysis was conducted using the Optima 8000 spectrometer. Electrochemical measurements were taken out on a CHI 760E electrochemial workstation. For product detection in electrochemical $CO_2$ reduction reaction ($CO_2$RR), the gaseous and liquid products were analyzed by Agilent 7890B gas chromatograph (Agilent, USA) and Bruker AVANCE III HD (BBO Probe, 300 MHz) nuclear magnetic resonance (Bruker, Germany), respectively.

### Synthesis of 2H-Cu nanocrystals (NCs)

The 2H-Cu NCs were synthesized *via* a one-pot wet-chemical method. In a typical experiment, 1.35 g of TOPO, 2.5 mL of OAm, and 8.5 μL HBr ($n_{Br}/n_{Cu}$ = ~ 1.25) were first added into a 50 mL three-neck flask. The as-obtained mixture was heated to 150 °C under vacuum and stirred for 10 min to remove water and low boiling point chemicals. After the temperature decreased to 120 °C and the flask was refilled with $N_2$ gas, 15.7 mg of $Cu(acac)_2$ were added and the solution color changed to green. Then the obtained mixture was degased again at 120 °C for 2 h, and the solution color changed from green to light yellow gradually. After that, the flowing $N_2$ gas was introduced into the flask and the temperature was increased to 150 °C. Subsequently, 0.6 mL of octylamine were injected into the flask with a syringe and the temperature was maintained for 10 min. Then, the mixture was heated to 320 °C under $N_2$ and this temperature was maintained for 50 min. The solution color changed from light yellow to brown to gray red gradually until the reaction completed. After the mixture was cooled down naturally to room temperature, 20 mL of hexane were added into the flask and the obtained gray blue mixture was centrifuged at 8000 rpm for 3 min. Finally the product was washed twice with 10 mL of hexane, and then re-dispersed in 2 mL of hexane for the further usage.

**Synthesis of *fcc*-Cu NCs**

The *fcc*-Cu NCs were prepared *via* a two-step wet-chemical method. In the first step for preparing the Cu seed solution, after 39.3 mg of $Cu(acac)_2$ and 5 mL of OAm were added into a three-neck flask, the mixture was heated to 150 °C under vaccum and stirred for 10 min. After $N_2$ was refilled into the flask, the mixture was heated to 230 °C which was maintained for 2 h prior to cooling down naturally to room temperature. Then, the obtained seed solution was sonicated under $N_2$ for 5 min before the further usage. In the second step, 50 μL of as-synthesized Cu seed solution were added into a 15 mL pressure tube containing 5 mL of OAm, 20 mg of AA and 39.3 mg of $Cu(acac)_2$. The aforementioned mixture was sonicated for 3 min and heated to 80 °C under vaccum and then stirred for 5 min. After the $N_2$ was refilled, the tube was sealed and transferred

into an oil-bath at 95 °C which was maintained for 12 h. After cooling down to room temperature, the obtained reddish solution was mixed with 20 mL hexane and sonicated for 5 min. The final product was collected by centrifugation at 8,000 rpm for 5 min, washed twice with hexane, and then re-dispersed in 20 mL of hexane for the further usage.

**Electrochemical $CO_2$RR measurements**

**Preparation of catalyst inks**

The amount of the catalyst was determined by the ICP-OES measurement. Typically, 500 μg of catalysts were dispersed into 200 μL of isopropanol, followed by the addition of 10 μL of Nafion solution (5 wt%). Then the resultant solution was sonicated in an ice-water bath for 30 min to obtain homogeneous catalyst ink. Subsequently, the catalyst ink was droped on the surface of glassy carbon electrode (GCE). The loading amount of catalysts was 400 μg $cm^{-2}$. The obtained working electrodes were dried under ambient conditions before test.

**Electrochemical $CO_2$RR measurements**

The electrochemical $CO_2$RR tests of 2H- and *fcc*-Cu NCs were performed in a typical gas-tight two-chamber H-type cell separated by an ion exchange membrane (Nafion 117). All electrochemical data were acquired on a CHI 760E workstation. All potentials were converted to reversible hydrogen electrode (RHE) scale based on the equation of E (*vs.* RHE) = E (*vs.* Ag/AgCl) + 0.197 V + 0.0591 × pH, in which no iR compensation was applied. In a typical three-electrode electrocatalytic system, the GCE with catalysts, Ag/AgCl (saturated KCl) electrode, and Pt foil were used as the working electrode, reference electrode in cathode chamber, and counter electrode in anode chamber, respectively. In a typical electrocatalytic $CO_2$RR test, 9 mL of 0.1 M $KHCO_3$ aqueous solution were added into the anode and cathode chambers, respectively. Then the electrolyte in cathode chamber was purged with $CO_2$ gas for 15 min before test. During

the electrochemical $CO_2$RR test, the electrolyte in cathode chamber was stirred at 300 rpm, and the $CO_2$ gas was bubbled into the cathode chamber at a flow rate of 20 standard cubic centimeters per minute (sccm).

The electrochemical $CO_2$RR was also performed in a three-channel flow cell composed of a gas-diffusion layer (GDL), an anion exchange membrane (Fumasep, FAA-3-PK-130) and a Ni foam (2 $cm^2$) anode. 1.0 M KOH aqueous solution was used as the electrolytes. Both electrolytes were circulated by a two-channel peristaltic pump (LongerPump, BT100-2J) with a flow rate of 20 mL $min^{-1}$. $CO_2$ gas at a flow rate of 20 sccm was continuously purged into the gas flow channel behind the GDL.

All the gaseous products of electrocatalytic $CO_2$RR were quantified by an on-line gas chromatography (GC, Agilent 7890B). The GC system is equipped with a thermal conductivity detector (TCD) and two flame ionization detectors (FIDs) as well. The TCD was used to quantify $H_2$ product. In parallel, two FIDs were utilized to analyze and quantify CO product or other hydrocarbon products, such as $CH_4$, $C_2H_4$, and $C_2H_6$. Each collection of GC spectrum took 9.5 mins, and three GC spectra were acquired at every applied potential. In addition, the resultant liquid products of electrocatalytic $CO_2$RR were collected and successively quantified by NMR characterization. In a typical experiment, 600 μL of the obtained electrolyte were mixed with 30 μL of $D_2O$ and 16.7 ppm (m/m) dimethyl sulfoxide (DMSO) as an internal standard. The $^1$H NMR spectra were acquired with water suppression using a pre-saturation approach. During the long-term electrocatalytic $CO_2$RR durability test, the gaseous products were collected by an automatic injection mode every half an hour, and the liquid products were collected every 2 hours, which was further quantified by NMR characterization based on the total quantity of electricity.

**Faradaic efficiency (FE) calculation**

The FEs of $CO_2$RR products were calculated based on the following equation,

$$FE = \frac{ZnF}{Q} \times 100\%,$$

where Z is the number of electrons when forming a specific product, *n* is the number of moles of the products, F is the Faradaic constant which equals to 96485, and Q is the amount of charges passed through the electrode during electrolysis.

The partial current density ($J_{partial}$) was calculated by the following equation,

$$J_{partial} = J_{total} \times FE,$$

where $J_{total}$ is the total geometric current density.

## *In situ* ATR-FTIR characterization

The *in situ* ATR-FTIR measurement was conducted on a Nicolet iS50 IR spectrometer based on the absorption mode equipped with a Mercury-Cadmium Telluride (MCT) detector cooled by liquid $N_2$. The working electrode for ATR-FTIR test was prepared by dropping the catalyst ink onto an Au-coated Si hemispherical prism (20 mm in diameter, MTI Corporation). The loading amounts of 2H- and *fcc*-Cu NCs were 200 μg $cm^{-2}$. The ATR-FTIR spectra were collected at a resolution of 8 $cm^{-1}$. The scan rate was 5 mV $s^{-1}$ with a time of expose of 10 s per spectrum.

## *In situ* DEMS characterization

The *in situ* DEMS patterns were collected on a Shanghai Linglu mass spectroscopy connected with a typical electrocatalytic cell containing $CO_2$-saturated 0.1 M $KHCO_3$ as electrolyte. The carbon paper loaded with catalysts, Ag/AgCl (saturated KCl) electrode and Pt wire were used as working electrode, reference electrode and counter electrode, respectively. The signals were collected during the linearly sweeping the working electrode potential from 0 to −1.60 V (*vs.* RHE) with a scanning rate of 5 mV $s^{-1}$ for three cycles.

**Density functional theory (DFT) calculations**

The DFT calculations were performed in the Vienna ab initio simulation packages (VASP),[1,2] using the generalized gradient approximation (GGA) with Perdew–Burke–Ernzerhof (PBE) parameterization.[3-5] The 2H-Cu (110), 2H-Cu (101), *fcc*-Cu (111), and *fcc*-Cu (100) surfaces were modeled as six-layer slabs with 2 × 2, 2 × 4, 3 × 3, and 3 × 3 supercells, respectively. The lower three layers were fixed and a vacuum space of 20 Å was set to eliminate possible interaction between the periodic images. A 3 × 3 × 1 Monkhorst–Pack k-point mesh was used for the Brillouin zone integration. A denser grid of 6 × 6 × 1 was used for electronic structure calculations. The energy cut-off of the plane wave basis was set as 520 eV. The convergence criteria of force and energy were set as 0.02 eV $Å^{-1}$ and $10^{-6}$ eV, respectively. The DFT-D3 method was applied to include the van der Waals interactions.[6] The Gibbs free energy ($\Delta G$) of the reaction intermediates is obtained by $\Delta G = \Delta \mathrm{E} + \Delta E_{\mathrm{ZPE}} - T\Delta S + \int C_P dT$, where $\Delta E$ is total energy difference, $\Delta E_{\mathrm{ZPE}}$ is the difference of the zero-point energy, $T$ is the temperature, $\Delta S$ is the difference of entropy, and $\int C_P dT$ is the heat capacity at 298.15 K. The computational results were post-processed by the VASPKIT code.[7] The transition states were simulated by using the climbing-image nudged elastic band (CI-NEB) method.[8]

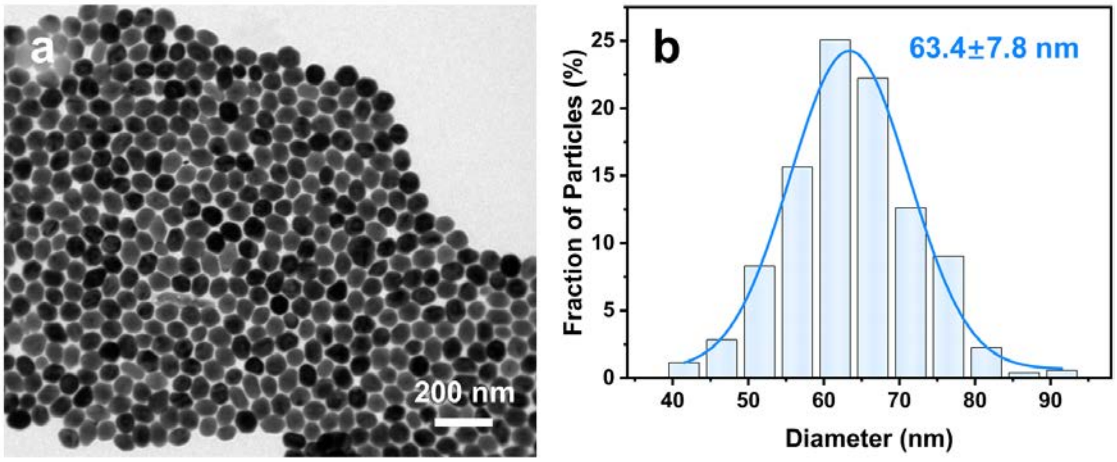


**Figure S1.** (a) Low-magnification TEM image, and (b) size distribution histogram of 2H-Cu NCs.

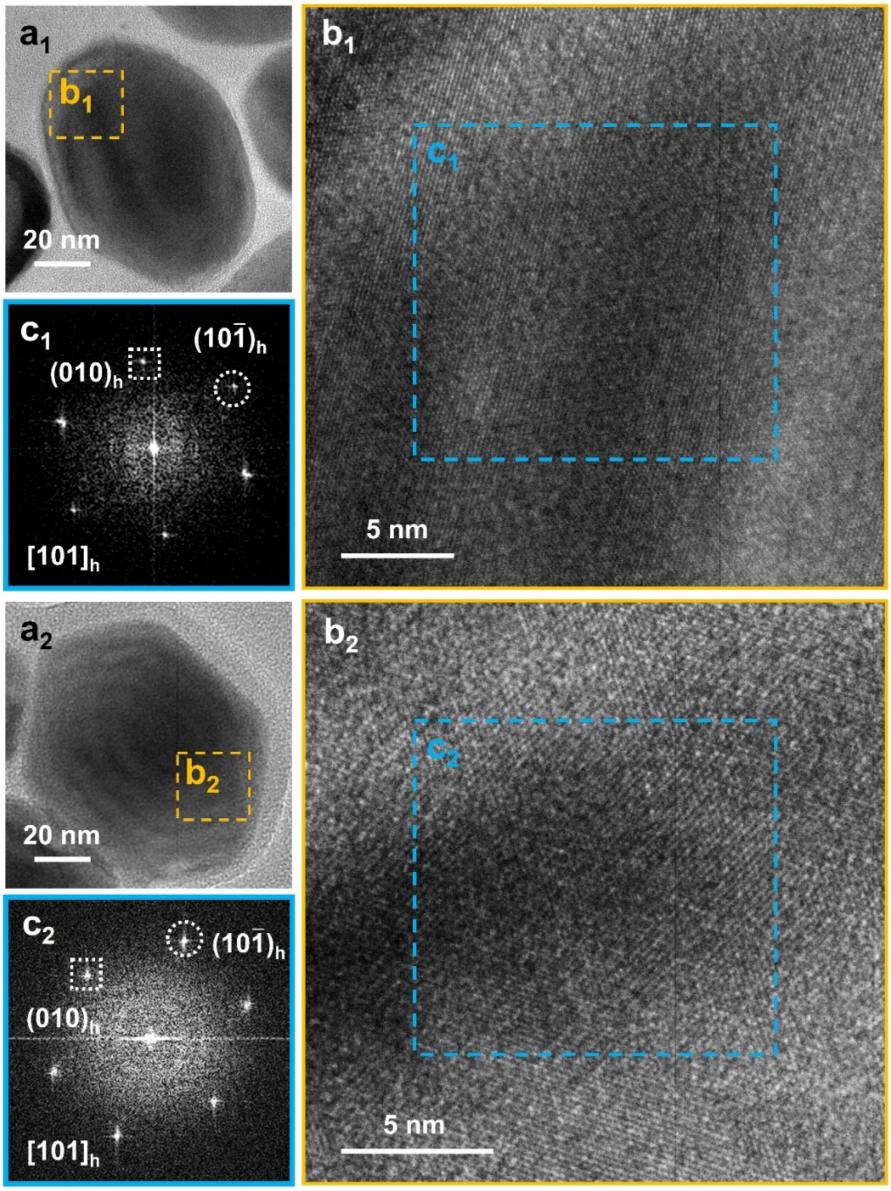


**Figure S2.** ($a_1$, $a_2$) High-resolution TEM (HRTEM) images of two different 2H-Cu NCs. ($b_1$, $b_2$) High-magnification HRTEM images from the selected areas in ($a_1$, $a_2$), respectively. ($c_1$, $c_2$) FFT patterns of the selected areas in ($b_1$, $b_2$), respectively.

Figure S2 exhibits two different 2H-Cu NCs which are not the same as that shown in Figure 1c. Both high-magnification HRTEM images (Figures S2$b_1$, S2$b_2$) and their corresponding FFT patterns (Figures S2$c_1$, S2$c_2$) show the typical facet, *i.e.*, 2H $(101)_h$, of the 2H phase, confirming the successful synthesis of unconventional 2H-Cu NCs.

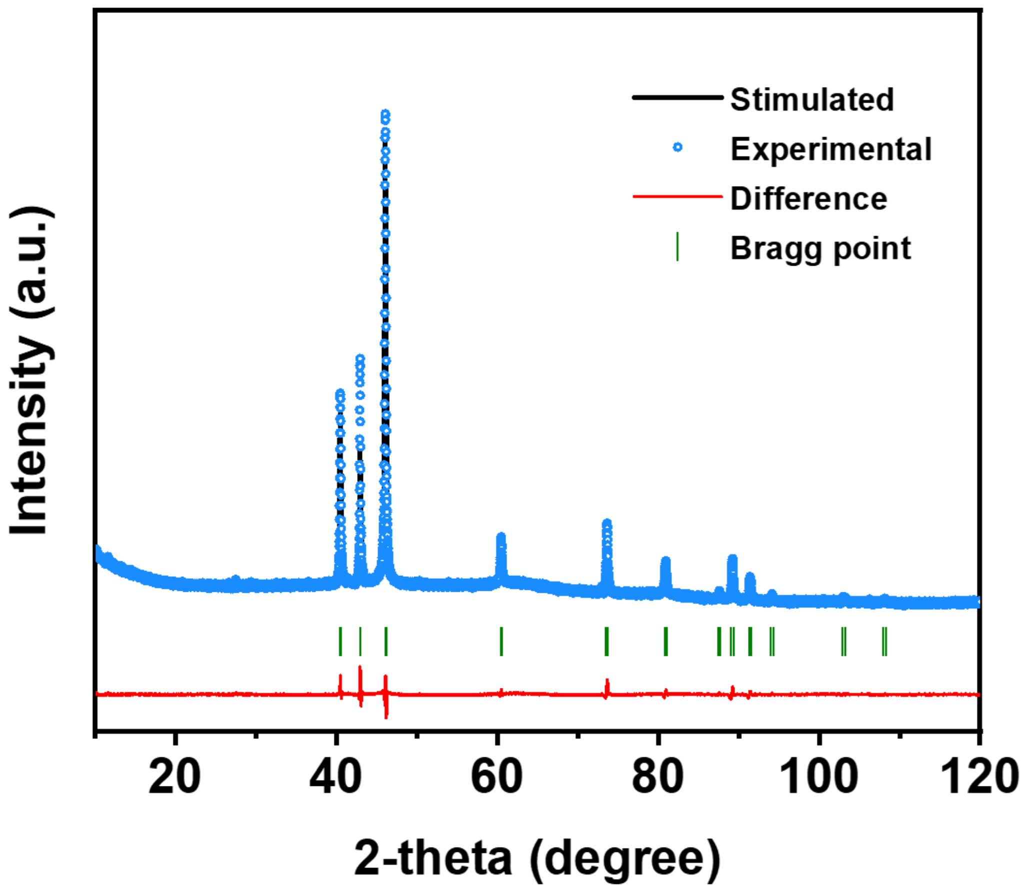


**Figure S3.** XRD pattern of 2H-Cu NCs obtained experimentally (blue) and through Rietveld refinement (black) according to the lattice parameters in Table S1.

Figure S3 demonstrates the agreement between the simulated result of the XRD Rietveld refinement and the experimental data. Additionally, the low R-pattern (Rp=3.1%) and R-weighted pattern (Rwp=5.2%) indicate a good fitting between the experimental data and computational model.

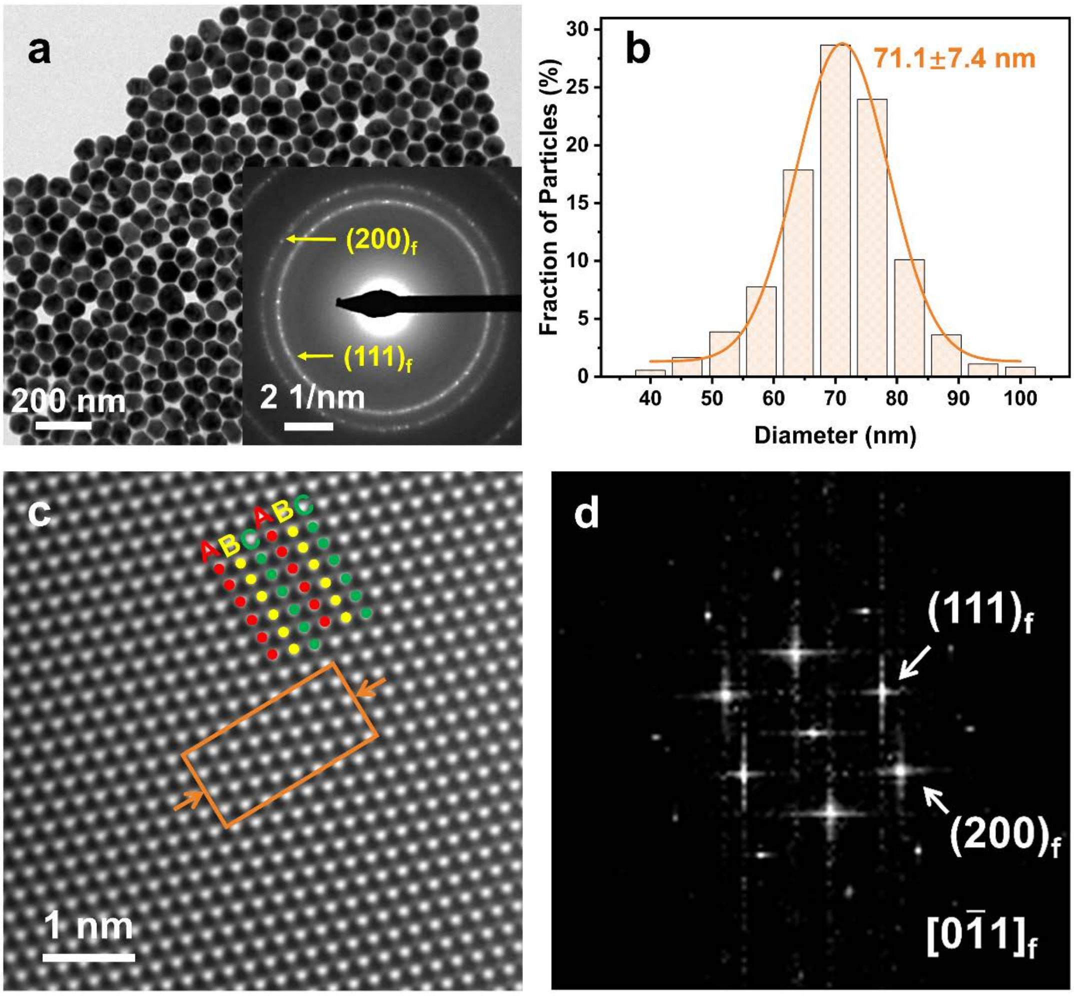


**Figure S4.** (a) TEM image, (b) size distribution histogram, and (c) spherical aberration-corrected HAADF-STEM image of *fcc*-Cu NCs. Inset in (a): the corresponding SAED pattern. (d) The corresponding FFT pattern of *fcc*-Cu NCs in (c).

Figure S4 displays a low-magnification TEM image (a) and the corresponding size distribution (b) of the *fcc*-Cu NCs. The SAED pattern (inset of (a)) and FFT pattern (d) show the *fcc* structure of Cu NCs. Additionally, the HAADF-STEM image (c) reveals a typical "ABC" stacking sequence along the $[111]_f$ close-packed direction, further confirming its *fcc* phase.

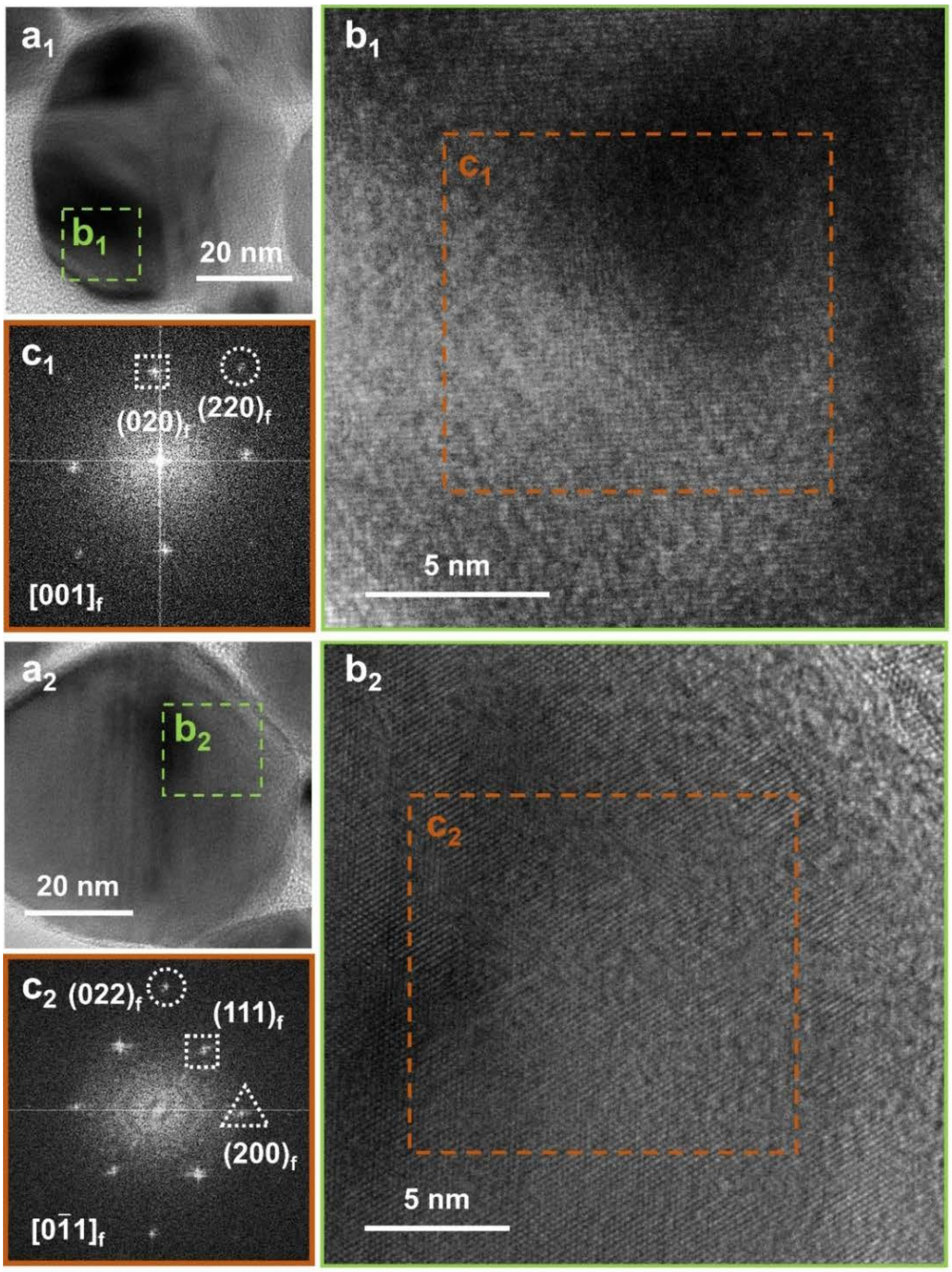


**Figure S5.** ($a_1$, $a_2$) HRTEM images of two different *fcc*-Cu NCs. ($b_1$, $b_2$) High-magnification HRTEM images from the selected areas in ($a_1$, $a_2$), respectively. ($c_1$, $c_2$) FFT patterns of the selected areas in ($b_1$, $b_2$), respectively.

Figure S5 shows two typical facets, *i.e.*, *fcc* $(100)_f$ (Figure S5$b_1$) and $(110)_f$ (Figure S5$b_2$) and their corresponding FFT patterns (Figures S5$c_1$, S5$c_2$), confirming the successful synthesis of conventional *fcc*-Cu NCs.

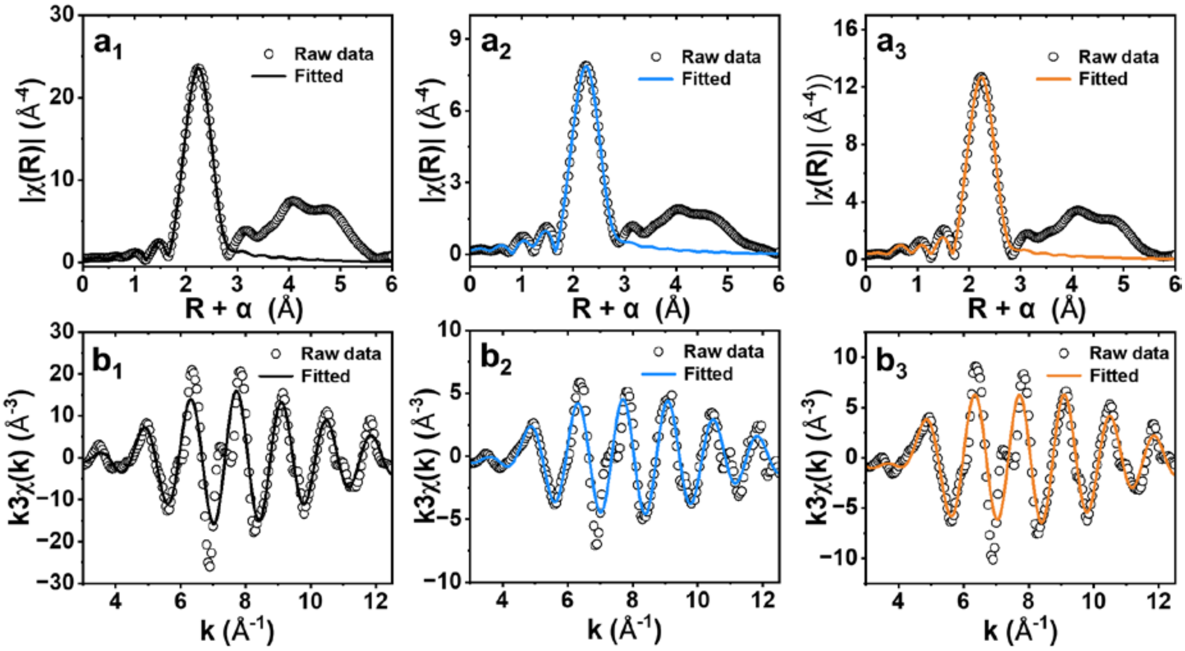


**Figure S6.** ($a_1$-$b_3$) Fitting results of EXAFS spectra at Cu K-edge for Cu foil ($a_1$, $b_1$), 2H-Cu NCs ($a_2$, $b_2$) and *fcc*-Cu NCs ($a_3$, $b_3$). ($a_1$-$a_3$) Cu K-edge EXAFS (points) and curve fitting (line) for Cu foil ($a_1$), 2H-Cu NCs ($a_2$) and *fcc*-Cu NCs ($a_3$) shown in *R*-space. The data are k3-weighted and no phase-corrected. ($b_1$-$b_3$) Cu K-edge EXAFS (points) and curve fitting (line) for Cu foil ($b_1$), 2H-Cu NCs ($b_2$) and *fcc*-Cu NCs ($b_3$) shown in $k^3$weighted *k*-space.

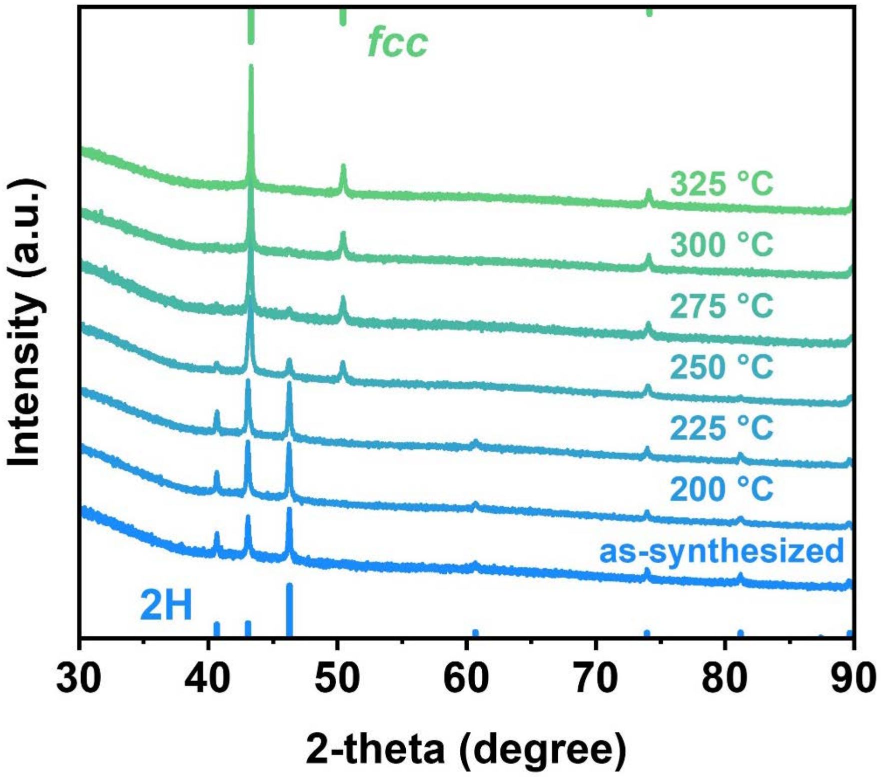


**Figure S7.** XRD patterns of 2H-Cu NCs annealed at different temperatures.

Figure S7 illustrates the gradual phase transition process of 2H-Cu NCs from the 2H to *fcc* phases as the annealing temperature increases.

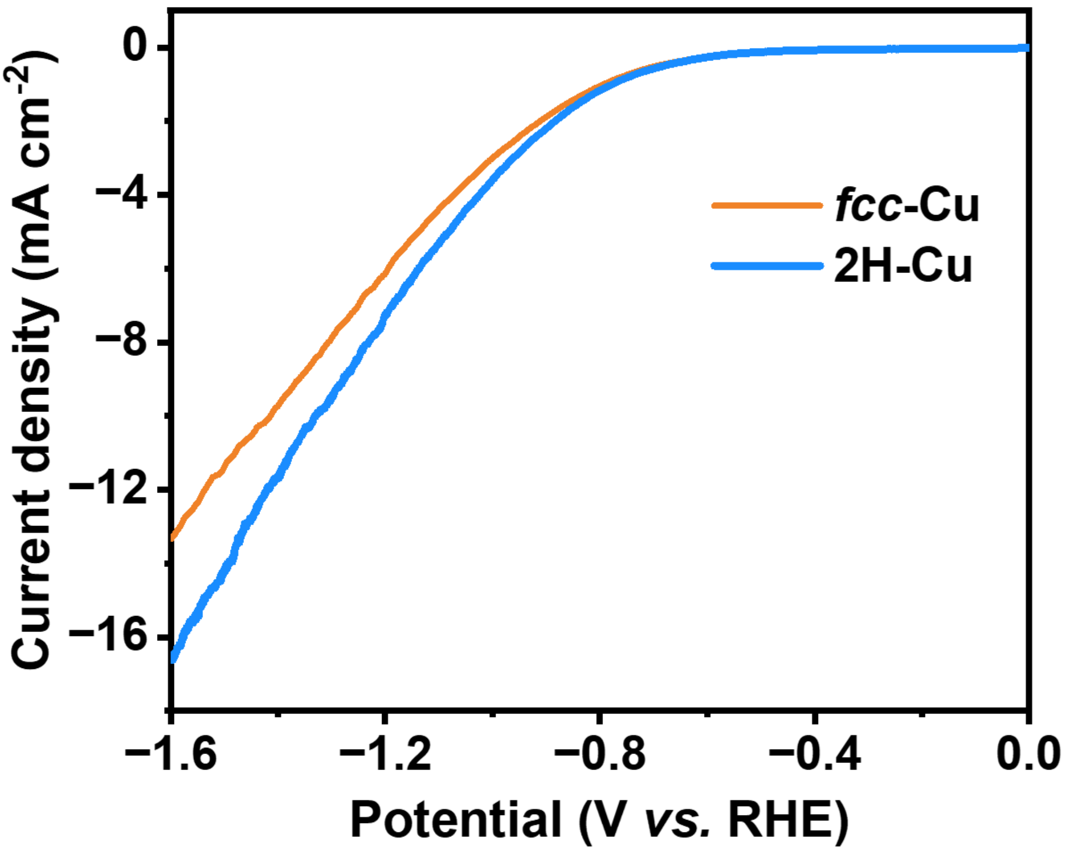


**Figure S8.** Linear sweep voltammetry (LSV) plots of 2H-Cu and *fcc*-Cu NCs at a scan rate of 10 mV $s^{-1}$ in a $CO_2$-saturated 0.1 M $KHCO_3$ aqueous solution.

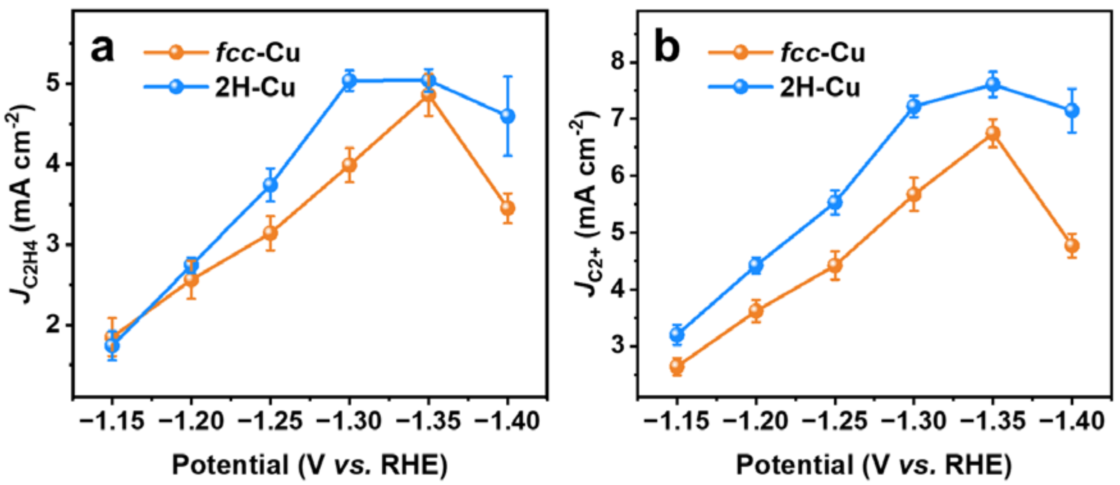


**Figure S9.** (a, b) The partial current densities of $C_2H_4$ (a) and $C_{2+}$ products (b) on 2H-Cu and *fcc*-Cu NCs under different potentials.

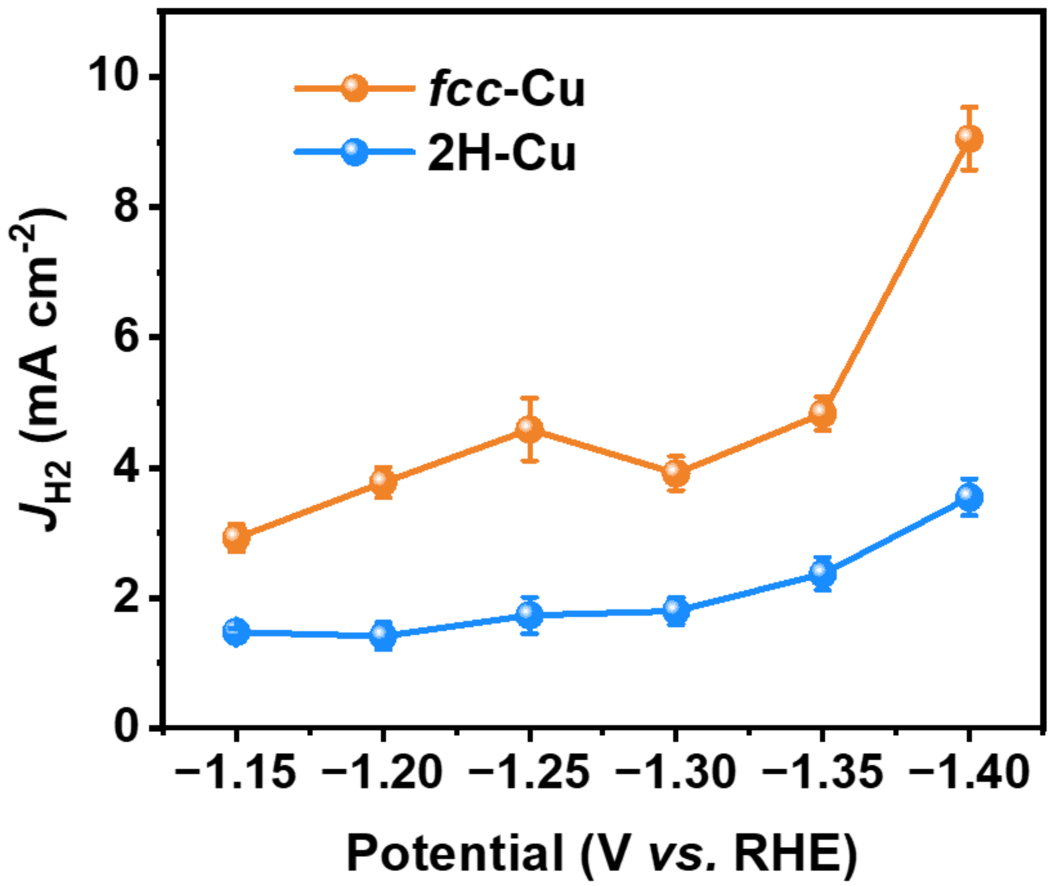


**Figure S10.** The partial current densities of $H_2$ product on 2H-Cu and *fcc*-Cu NCs under different potentials.

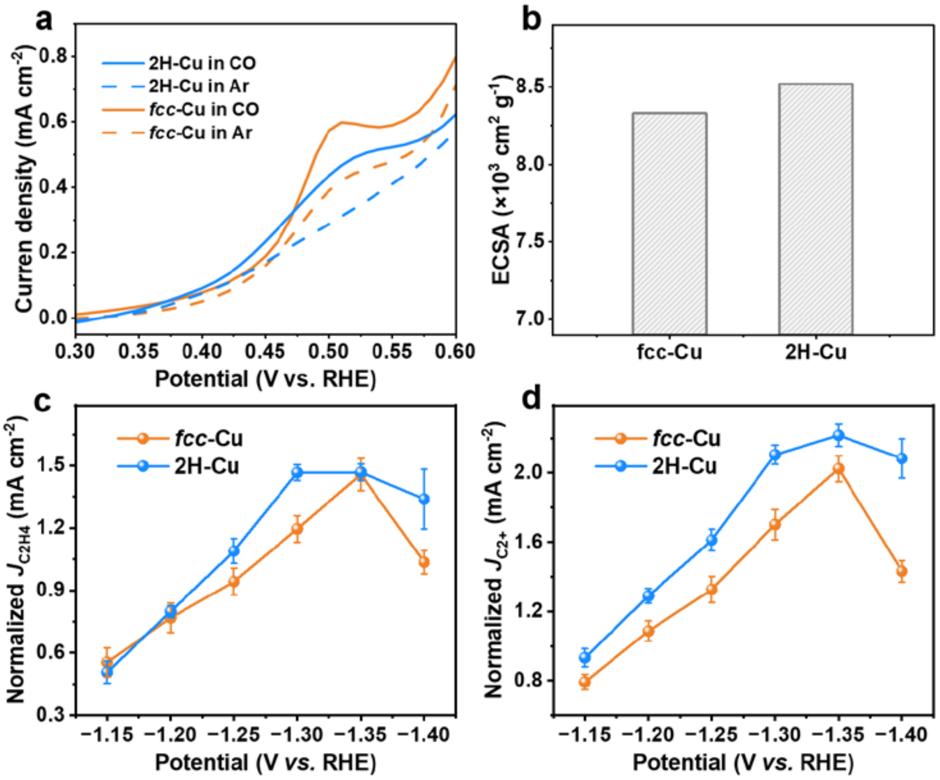


**Figure S11.** (a) The electrochemical CO stripping voltammetry tests of *fcc*-Cu, and 2H-Cu NCs in 0.1 M $KHCO_3$ electrolyte. (b) ECSAs of *fcc*-Cu (8.33×10$^3$ cm$^2$ g$^{-1}$), and 2H-Cu NCs (8.52×10$^3$ cm$^2$ g$^{-1}$) calculated based on (a). (c, d) The normalized partial current densities of $C_2H_4$ (c) and $C_{2+}$ products (d) on *fcc*-Cu, and 2H-Cu NCs under different potentials.

Figure S11 illustrates the determination of electrochemically active surface area (ECSA) using CO stripping voltammetry, and the corresponding ECSA-normalized partial current densities for $C_2H_4$ and total $C_{2+}$ products on the *fcc*-Cu and 2H-Cu NCs. Figure S11a was obtained by linear sweep voltammetry under CO or Ar atmosphere using an electrode loaded with 0.2 mg of catalysts at a scan rate of 10 mV s$^{-1}$. Figure S11b was calculated using the following equation, ECSA= $Q_{CO}$ / ($m_{catalyst}$ × 420 μC cm$^{-2}$), where $Q_{CO}$ is obtained by integrating the peak area in (a) from 0.45 to 0.55 V and $m_{catalyst}$ is the loading mass of catalyst. Figures S11c and d were derived by normalizing the partial current densities using the ECSA values in (b).

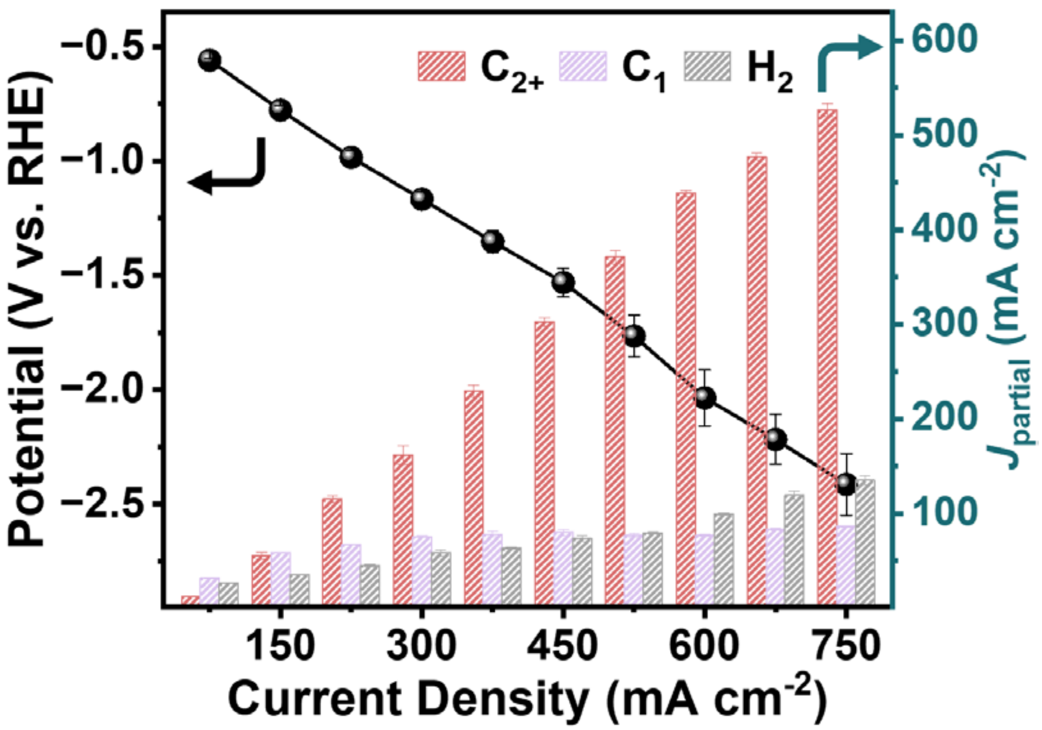


**Figure S12.** The partial current densities ($J_{partial}$) of products and the corresponding potentials of different current densities on 2H-Cu NCs in 1 M KOH electrolytes in a flow cell.

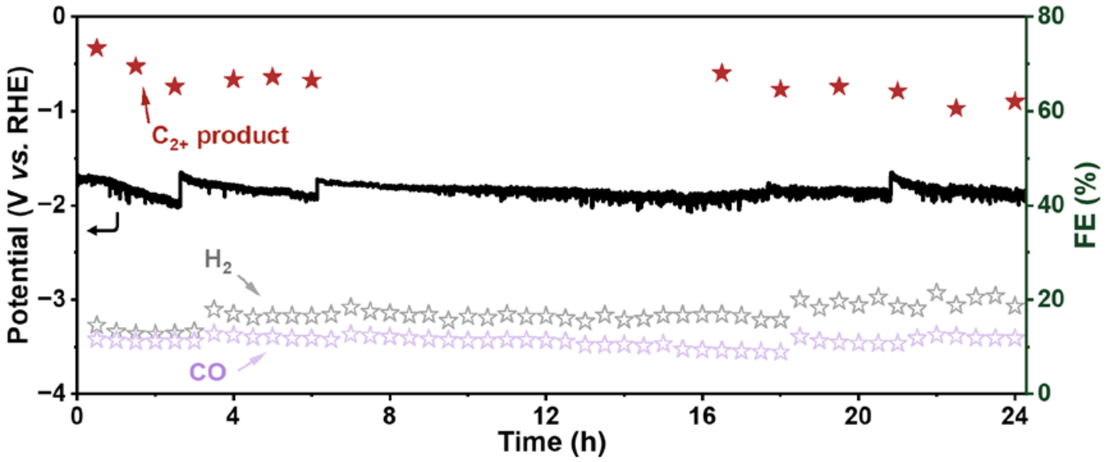


**Figure S13.** The long-term stability test of 2H-Cu NCs in a flow cell at a current density of 500 mA $cm^{-2}$.

Compared to Figure 3e, Figure S13 illustrates FEs of more products, including $H_2$, CO, and all $C_{2+}$ products, during the long-term test.

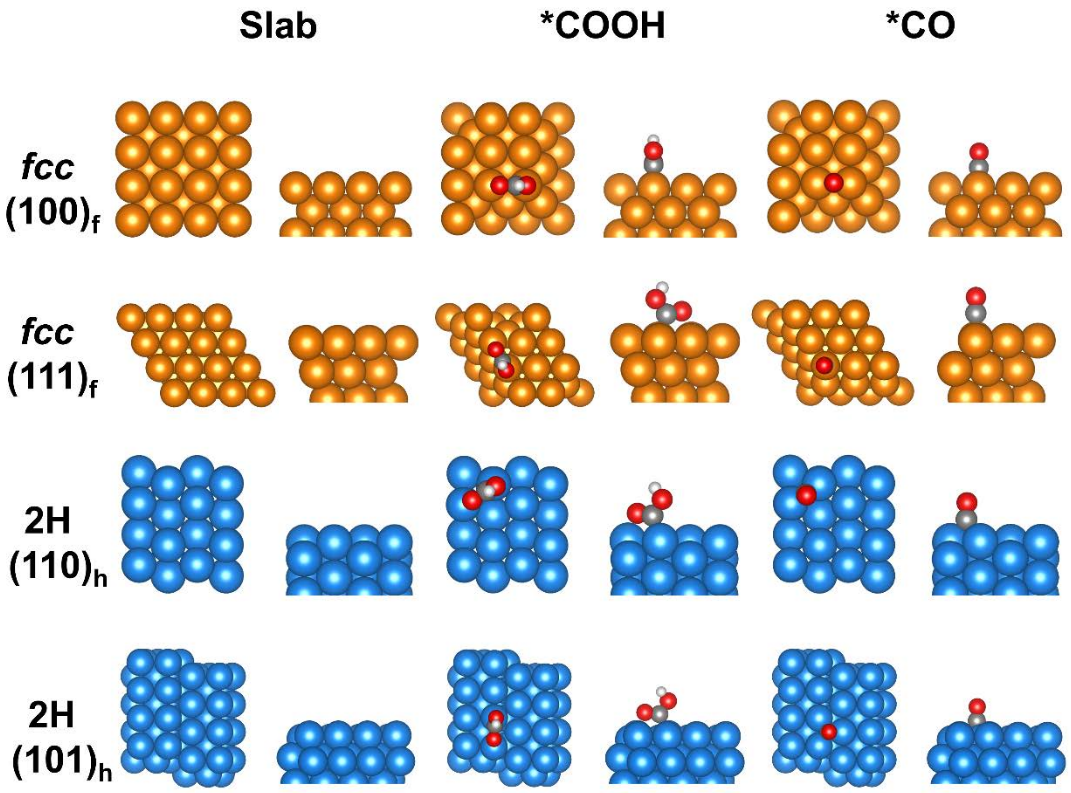


**Figure S14.** Top and side views of the optimized schematic models of the *CO conversion process, which show the clean, *COOH-, and *CO-adsorbed *fcc*-Cu $(100)_f$, *fcc*-Cu $(111)_f$, 2H-Cu $(110)_h$, and 2H-Cu $(101)_h$ facets.

Figure S14 illustrates the detailed structural conversion of $CO_2$ to *CO on the *fcc*-Cu $(100)_f$, *fcc*-Cu $(111)_f$, 2H-Cu $(110)_h$, and 2H-Cu $(101)_h$ facets, presented in both top and side views.

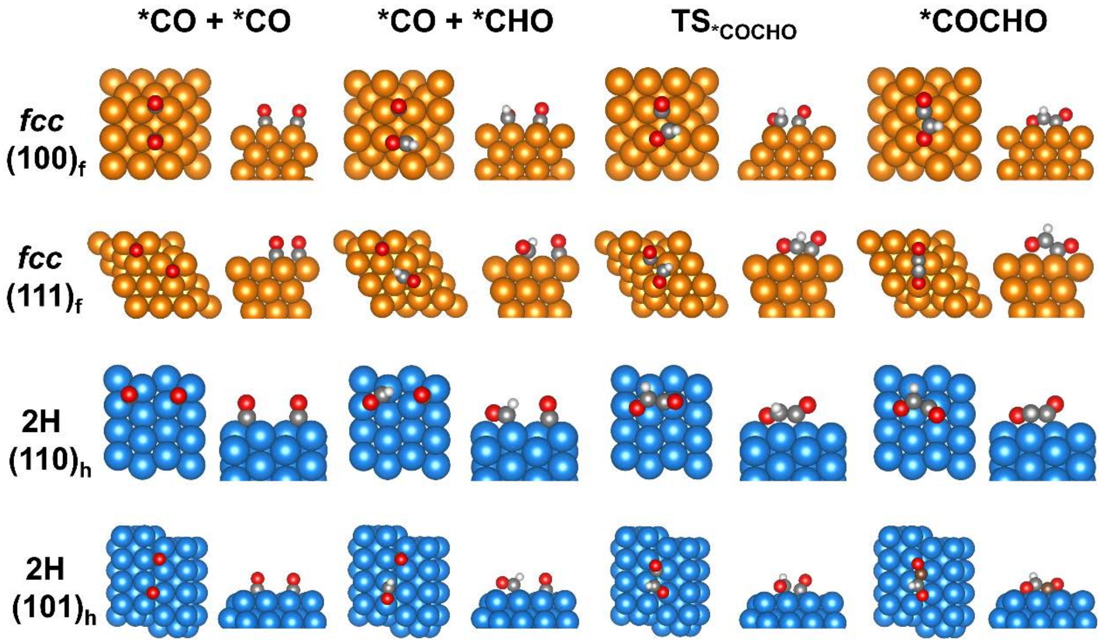


**Figure S15.** Top and side views of the optimized schematic models of the C−C coupling process on *fcc*-Cu $(100)_f$, *fcc*-Cu $(111)_f$, 2H-Cu $(110)_h$, and 2H-Cu $(101)_h$ facets.

Figure S15 shows the detailed structural conversion of C−C coupling process, transitioning from *CO to *COCHO, on the *fcc*-Cu $(100)_f$, *fcc*-Cu $(111)_f$, 2H-Cu $(110)_h$, and 2H-Cu $(101)_h$ facets, presented in both top and side views.

**Table S1.** Crystallographic parameters of modeled 2H-Cu.

| | |
|---|---|
| Space group | $P6_3/mmc$ |
| *a* [Å] | 2.57441(3) |
| *b* [Å] | 2.57441(3) |
| *c* [Å] | 4.21417(8) |
| $\alpha$ [deg] | 90 |
| $\beta$ [deg] | 90 |
| $\gamma$ [deg] | 120 |

**Table S2.** Local structure parameters for Cu K-edge EXAFS fitting for Cu foil, 2H-Cu NCs, and *fcc*-Cu NCs.

| Sample | Shell | N | R [Å] | $\Delta E_0$ (eV) | $\sigma^2$ [$10^{-3}$Å$^2$] | R-factor ($10^{-2}$) |
|---|---|---|---|---|---|---|
| **Cu foil** | Cu-Cu | 12 | 2.54 ± 0.003 | 4.09 ± 0.46 | 8.55 ± 0.37 | 0.12 |
| **2H-Cu** | Cu-Cu | 7.11 ± 1.03 | 2.55 ± 0.009 | 4.73 ± 1.35 | 6.53 ± 0.12 | 0.16 |
| ***fcc*-Cu** | Cu-Cu | 8.88 ± 1.55 | 2.54 ± 0.011 | 4.05 ± 1.68 | 7.91 ± 0.16 | 0.39 |

$S_0^2$ was fixed as 0.85, which was determined by Cu foil,

N = coordination number,

R = distance between absorber and backscatter atoms,

$\sigma^2$ = Debye-Waller factor to account for both thermal and structural disorders,

R-factor indicates the goodness of the fit.

**Table S3.** Comparison of the $CO_2$RR performance of 2H-Cu NCs in this work with the previously reported representative Cu-based electrocatalysts in H-type cells.

| **Cu-based electrocatalysts** | **Potential (*vs.* RHE)** | **Main product FE** | | **Electrolyte** | **Reference** |
|---|---|---|---|---|---|
| | | **$C_2H_4$** | **$C_{2+}$** | | |
| **2H-Cu NCs** | **−1.30 V (no iR compensation)** | **50.3%** | **72.4%** | **0.1 M $KHCO_3$** | **This Work** |
| *fcc*-Cu NCs | −1.35 V (no iR compensation) | 38.6% | 53.6% | 0.1 M $KHCO_3$ | This Work |
| 4H Au@Cu nanoribbons | −1.11 V | 44.9% | - | 0.1 M $KHCO_3$ | 9 |
| Polycrystalline Cu foil | −1.10 V | 54.5% | - | 0.1 M $KHCO_3$ | 10 |
| electrochemically treated Cu foil | −1.00 V | - | 65% | 0.1 M $KHCO_3$ | 11 |
| Star decahedron Cu nanoparticles | −0.99 V | 52.4% | - | 0.1 M $KHCO_3$ | 12 |
| Anodic-treated Cu | −1.0 V | 45% | 76% | 0.1 M $KHCO_3$ | 13 |
| Cu nanowires with rich surface step | −1.01 V | 77.4% | - | 0.1 M $KHCO_3$ | 14 |
| High facet of wrinkled Cu | −1.2 V | 34.0% | 57.1% | 0.1 M $KHCO_3$ | 15 |
| Boundary-abundant $Cu_2O$-Cu nanocubes | −1.0 V | 55.2% | 68.2% | 0.1 M $KHCO_3$ | 16 |
| $Cu_2O$ superparticle-CP3 | −1.15 V | 53.2% | 74.2% | 0.1 M $KHCO_3$ | 17 |
| High-index facets of Cu | −1.09 V | - | 72.7% | 0.1 M $KHCO_3$ | 18 |
| Oxide-derived Cu | −0.55 V | 38.6% | 75.6% | 4 M NaCl | 19 |
| Cu-on-$Cu_3N$ | −0.95 V | 39 ± 2% | 64 ± 2% | 0.1 M $KHCO_3$ | 20 |
| $Cu_xC_yO_z$ | −1.0 V | - | 70.3% | 0.1 M $KHCO_3$ | 21 |
| B-doped Cu | −1.1 V | 52 ± 2% | 79 ± 2% | 0.1 M $KHCO_3$ | 22 |
| CuO nanosheets | −0.99 V | 27.0% | 34.7% | 0.1 M | 23 |

| | | | | | |
|---|---|---|---|---|---|
| | | | | $KHCO_3$ | |
| $CuO_x$ nanocubes | −0.97 V | 34% | 48% | 0.1 M $KHCO_3$ | 24 |
| $Cu_2S$@Cu nanoparticles with vacancy | −1.1 V | 42.3% | 46.7% | 0.1 M $KHCO_3$ | 25 |
| Electro-redeposition Cu foil | −1.2 V | 38% | 52% | 0.1 M $KHCO_3$ | 26 |
| Cu/$Cu_{2+1}$O/NC | −1.1 V | 48.3% | - | 0.1 M $KHCO_3$ | 27 |
| PTFE-treated electrodeposited Cu | -1.25 V | 67.3% | - | 0.1 M CsI | 28 |
| CuO/F/C(w) | −1.4 V | 39.9 % | 56.8 % | 0.5 M $KHCO_3$ | 29 |
| $Cu_2O$ cube | −1.01 V | 23.2% | 43.7% | 0.1 M $KHCO_3$ | 30 |
| $SnO_x$-Cu | −1.3 V | - | 66.3% | 0.1 M $KHCO_3$ | 31 |

Abbreviations: $Cu_2O$ superparticle-CP3, $Cu_2O$ superparticle pre-electroreduced by chronopotentiometry method at a cathodic current density of −3 mA $cm^{-2}$; Cu/$Cu_{2+1}$O/NC: Cu, CuO, and $Cu_2O$ nanocrystals; CuO/F/C(w): core–shell CuO nanospheres, carbon nanotubes (CNT), and polytetrafluoroethylene (PTFE) are integrated into a wire structure.

**Table S4.** Comparison of the $CO_2$RR performance of 2H-Cu NCs in this work with the previously reported representative Cu-based electrocatalysts in flow cells.

| **Cu-based electrocatalysts** | **Main product FE** | | **$J_{C2+}$ (mA cm$^{-2}$) (maximum)** | **Electrolyte** | **Reference** |
|---|---|---|---|---|---|
| | **$C_2H_4$** | **$C_{2+}$** | | | |
| **2H-Cu NCs** | **51.7%** | **73.0%** | **526** | **1 M KOH** | **This Work** |
| *fcc*-2H-*fcc* Au–Cu Janus nanostructures | - | 61.3% | 220.8 | 1 M KOH | 32 |
| $CuN_3$-Ag nanocubes | 36.6% | 52.5% | 38.3 | 1 M KOH | 33 |
| p-Cu@m-$SiO_2$ | 56% | - | 212 | 1 M KOH | 34 |
| cAA-CuNW | 60.7% | - | 539 | 1 M KOH | 35 |
| Cu NCs/$Al_2O_3$ -10C | 60.4% | - | 181.2 | 5 M KOH | 36 |
| $V_{tri\text{-}Cu}$-rich-Cu | ~50% | 81.3% | 754 | 1 M KOH | 37 |
| Cu nanocrystals (pulsed electrolysis) | 44% | 63.6% | 161.5 | 1 M KOH | 38 |
| 5% Cu–$CeO_2$ nanorod | 46.2% | 58.5% | 146.2 | 1 M KOH | 39 |
| 3-shell Cu HoMSs | - | 77% | 513.7 | 0.5 M $KHCO_3$ | 40 |
| P-$Cu_{1.65}$/$Cu_2$OF | 36.6% | 80.2% | 288 | 1 M KOH | 41 |
| Ionomer films modified Cu | - | 75% | 225 | 1 M $KHCO_3$ | 42 |
| Hierarchical cavity Cu | - | 78.7% | 236.1 | 1 M KOH | 43 |
| Cu-$Pt_1$ | - | 71.8% | 143.6 | 1 M KOH | 44 |
| $Cu_xO$-$CeO_2$ | 51.7% | 80% | 160 | 1 M KOH | 45 |

Abbreviations: p-Cu@m-$SiO_2$, porous-Cu@mesoporous-$SiO_2$ composite; cAA-CuNW, Cu nanowire with ascorbic acid nanoconfined by graphene quantum dots; Cu NCs/$Al_2O_3$-10C, Cu nanocrystal treated by 10 cycles of ALD $Al_2O_3$; $V_{tri\text{-}Cu}$-rich-Cu, triple-copper-vacancy associates with different concentrations were deliberately introduced on the Cu nanowires; 3-shell Cu HoMSs, $Cu_2O$ hollow multi-shell structures; P-$Cu_{1.65}$/$Cu_2$OF, F-stabilized Cu(I)/Cu(0) catalyst constructed by a pulsed potential conversion process; Cu-$Pt_1$, Pt single-atoms modified Cu catalysts.

## References


1. Kresse, G.; Furthmuller, J., Efficient iterative schemes for ab initio total-energy calculations using a plane-wave basis set. *Phys. Rev. B* **1996**, *54*, 11169-11186.
2. Kresse, G.; Furthmuller, J., Efficiency of ab-initio total energy calculations for metals and semiconductors using a plane-wave basis set. *Comp. Mater. Sci.* **1996**, *6*, 15-50.
3. Perdew, J.; Burke, K.; Ernzerhof, M., Generalized gradient approximation made simple. *Phys. Rev. Lett.* **1996**, *77*, 3865-3868.
4. Blochl, P., Projector augmented-wave method. *Phys Rev B* **1994**, *50*, 17953-17979.
5. Kresse, G.; Joubert, D., From ultrasoft pseudopotentials to the projector augmented-wave method. *Phys. Rev. B* **1999**, *59*, 1758-1775.
6. Grimme, S.; Antony, J.; Ehrlich, S.; Krieg, H., A consistent and accurate ab initio parametrization of density functional dispersion correction (DFT-D) for the 94 elements H-Pu. *J. Chem. Phys.* **2010**, *132*, 154104.
7. Wang, V.; Xu, N.; Liu, J.; Tang, G.; Geng, W., VASPKIT: A user-friendly interface facilitating high-throughput computing and analysis using VASP code. *Comput. Phys. Commun.* **2021**, *267*, 108033.
8. Henkelman, G.; Uberuaga, B.; Jónsson, H., A climbing image nudged elastic band method for finding saddle points and minimum energy paths. *J. Chem. Phys.* **2000**, *113*, 9901-9904.
9. Chen, Y.; Fan, Z.; Wang, J.; Ling, C.; Niu, W.; Huang, Z.; Liu, G.; Chen, B.; Lai, Z.; Liu, X.; Li, B.; Zong, Y.; Gu, L.; Wang, J.; Wang, X.; Zhang, H., Ethylene selectivity in electrocatalytic $CO_2$ reduction on Cu nanomaterials: a crystal phase-dependent study. *J. Am. Chem. Soc.* **2020**, *142*, 12760-12766.
10. Li, Z.; Wang, L.; Wang, T.; Sun, L.; Yang, W., Steering the dynamics of reaction intermediates and catalyst surface during electrochemical pulsed $CO_2$ reduction for enhanced $C_{2+}$ selectivity. *J. Am. Chem. Soc.* **2023**, *145*, 20655-20664.
11. Zhan, C.; Dattila, F.; Rettenmaier, C.; Herzog, A.; Herran, M.; Wagner, T.; Scholten,

F.; Bergmann, A.; Lopez, N.; Roldan Cuenya, B., Key intermediates and Cu active sites for $CO_2$ electroreduction to ethylene and ethanol. *Nat. Energy* **2024**, *9*, 1485-1496.

12. Choi, C.; Cheng, T.; Flores Espinosa, M.; Fei, H.; Duan, X.; Goddard, W.; Huang, Y., A highly active star decahedron Cu nanocatalyst for hydrocarbon production at low overpotentials. *Adv. Mater.* **2019**, *31*, e1805405.

13. Arán-Ais, R. M.; Scholten, F.; Kunze, S.; Rizo, R.; B., R. C., The role of in situ generated morphological motifs and Cu(I) species in $C_{2+}$ product selectivity during $CO_2$ pulsed electroreduction. *Nat. Energy* **2020**, *5*, 317-325.

14. Choi, C.; Kwon, S.; Cheng, T.; Xu, M. J.; Tieu, P.; Lee, C.; Cai, J.; Lee, H. M.; Pan, X. Q.; Duan, X. F.; Goddard, W. A.; Huang, Y., Highly active and stable stepped Cu surface for enhanced electrochemical $CO_2$ reduction to $C_2H_4$. *Nat. Catal.* **2020**, *3*, 804-812.

15. Kim, J.; Park, W.; Choi, C.; Kim, G.; Cho, K.; Lim, J.; Kim, S.; Al-Saggaf, A.; Gereige, I.; Lee, H.; Jung, W.; Jung, Y.; Jung, H., High facets on nanowrinkled Cu via chemical vapor deposition graphene growth for efficient $CO_2$ reduction into ethanol. *ACS Catal.* **2021**, *11*, 5658-5665.

16. Wu, Q.; Du, R.; Wang, P.; Waterhouse, G.; Li, J.; Qiu, Y.; Yan, K.; Zhao, Y.; Zhao, W.; Tsai, H.; Chen, M.; Hung, S.; Wang, X.; Chen, G., Nanograin-boundary-abundant $Cu_2O$-Cu nanocubes with high $C_{2+}$ selectivity and good stability during electrochemical $CO_2$ reduction at a current density of 500 mA/cm$^2$. *ACS Nano* **2023**, *17*, 12884-12894.

17. Jiang, Y.; Wang, X.; Duan, D.; He, C.; Ma, J.; Zhang, W.; Liu, H.; Long, R.; Li, Z.; Kong, T.; Loh, X. J.; Song, L.; Ye, E.; Xiong, Y., Structural reconstruction of $Cu_2O$ superparticles toward electrocatalytic $CO_2$ reduction with high $C_{2+}$ products selectivity. *Adv. Sci.* **2022**, *9*, 2105292.

18. Lei, Q.; Zhu, H.; Song, K.; Wei, N.; Liu, L.; Zhang, D.; Yin, J.; Dong, X.; Yao, K.; Wang, N.; Li, X.; Davaasuren, B.; Wang, J.; Han, Y., Investigating the origin of enhanced $C_{2+}$ selectivity in oxide-/hydroxide-derived copper electrodes during $CO_2$ electroreduction. *J. Am. Chem. Soc.* **2020**, *142*, 4213-4222.

19. Li, J.; Xu, K.; Liu, F.; Li, Y.; Hu, Y.; Chen, X.; Wang, H.; Xu, W.; Ni, Y.; Ding, G.; Zhao, T.; Yu, M.; Xie, W.; Cheng, F., Hollow hierarchical $Cu_2O$-derived electrocatalysts steering $CO_2$ reduction to multi-carbon chemicals at low overpotentials. *Adv. Mater.* **2023**, *35*, 2301127.

20. Liang, Z.; Zhuang, T.; Seifitokaldani, A.; Li, J.; Huang, C.; Tan, C.; Li, Y.; De Luna, P.; Dinh, C.; Hu, Y.; Xiao, Q.; Hsieh, P.; Wang, Y.; Li, F.; Quintero-Bermudez, R.; Zhou, Y.; Chen, P.; Pang, Y.; Lo, S. C.; Chen, L.; Tan, H.; Xu, Z.; Zhao, S.; Sinton, D.; Sargent, E. H., Copper-on-nitride enhances the stable electrosynthesis of multi-carbon products from $CO_2$. *Nat. Commun.* **2018**, *9*, 3828.

21. Xu, L.; Feng, J.; Wu, L.; Song, X.; Tan, X.; Zhang, L.; Ma, X.; Jia, S.; Du, J.; Chen, A.; Sun, X.; Han, B., Identifying the optimal oxidation state of Cu for electrocatalytic reduction of $CO_2$ to $C_{2+}$ products. *Green Chem.* **2023**, *25*, 1326-1331.

22. Zhou, Y.; Che, F.; Liu, M.; Zou, C.; Liang, Z.; De Luna, P.; Yuan, H.; Li, J.; Wang, Z.; Xie, H.; Li, H.; Chen, P.; Bladt, E.; Quintero-Bermudez, R.; Sham, T. K.; Bals, S.; Hofkens, J.; Sinton, D.; Chen, G.; Sargent, E. H., Dopant-induced electron localization drives $CO_2$ reduction to $C_2$ hydrocarbons. *Nat. Chem.* **2018**, *10*, 974-980.

23. Wang, X.; Klingan, K.; Klingenhof, M.; Moller, T.; Ferreira de Araujo, J.; Martens, I.; Bagger, A.; Jiang, S.; Rossmeisl, J.; Dau, H.; Strasser, P., Morphology and mechanism of highly selective Cu(II) oxide nanosheet catalysts for carbon dioxide electroreduction. *Nat. Commun.* **2021**, *12*, 794.

24. Moller, T.; Scholten, F.; Thanh, T. N.; Sinev, I.; Timoshenko, J.; Wang, X.; Jovanov, Z.; Gliech, M.; Roldan Cuenya, B.; Varela, A. S.; Strasser, P., Electrocatalytic $CO_2$ reduction on $CuO_x$ nanocubes: tracking the evolution of chemical state, geometric structure, and catalytic selectivity using operando spectroscopy. *Angew. Chem. Int. Ed.* **2020**, *59*, 17974-17983.

25. Zhuang, T.; Liang, Z.; Seifitokaldani, A.; Li, Y.; De Luna, P.; Burdyny, T.; Che, F.; Meng, F.; Min, Y.; Quintero-Bermudez, R.; Dinh, C.; Pang, Y.; Zhong, M.; Zhang, B.; Li, J.; Chen, P.; Liang, H.; Ge, W.; Ye, B.; Sinton, D.; Yu, S.; Sargent, E. H., Steering

post-C-C coupling selectivity enables high efficiency electroreduction of carbon dioxide to multi-carbon alcohols. *Nat. Catal.* **2018**, *1*, 421-428.
26. De Luna, P.; Quintero-Bermudez, R.; Dinh, C.; Ross, M.; Bushuyev, O.; Todorović, P.; Regier, T.; Kelley, S.; Yang, P.; Sargent, E. H., Catalyst electro-redeposition controls morphology and oxidation state for selective carbon dioxide reduction. *Nat. Catal.* **2018**, *1*, 103-110.
27. Tong, X.; Zhang, P.; Chen, P.; He, Z.; Kang, X.; Yin, Y.; Cheng, Y.; Zhou, M.; Jing, L.; Wang, C.; Xu, B.; Zheng, L.; Xing, X.; Wu, Z.; Han, B., Switching $CO_2$ electroreduction pathways between ethylene and ethanol via tuning microenvironment of the coating on copper nanofibers. *Angew. Chem. Int. Ed.* **2024**, *64*, e202413005.
28. Deng, T.; Jia, S.; Chen, C.; Jiao, J.; Chen, X.; Xue, C.; Xia, W.; Xing, X.; Zhu, Q.; Wu, H.; He, M.; Han, B., Polymer modification strategy to modulate reaction microenvironment for enhanced $CO_2$ electroreduction to ethylene. *Angew. Chem. Int. Ed.* **2024**, *63*, e202313796.
29. Li, Y.; Pei, Z.; Luan, D.; Lou, X., Superhydrophobic and conductive wire membrane for enhanced $CO_2$ electroreduction to multicarbon products. *Angew. Chem. Int. Ed.* **2023**, *62*, e202302128.
30. Choi, W.; Chae, Y.; Liu, E.; Kim, D.; Drisdell, W.; Oh, H.; Koh, J.; Lee, D.; Lee, U.; Won, D., Exploring the influence of cell configurations on Cu catalyst reconstruction during $CO_2$ electroreduction. *Nat. Commun.* **2024**, *15*, 8345.
31. Ding, S.; Su, Q.; Jin, Z.; Fan, J.; Dong, Y.; Liu, L.; Han, T.; Xinyu, E.; Wang, J.; Qi, K.; Cui, X., Atomically engineered $SnO_x$-Cu interfacial sites regulate water dissociation toward highly selective $CO_2$ to $C_2$ conversion. *Adv. Funct. Mater.* **2025**, e28796.
32. Ma, Y.; Sun, M.; Xu, H.; Zhang, Q.; Lv, J.; Guo, W.; Hao, F.; Cui, W.; Wang, Y.; Yin, J.; Wen, H.; Lu, P.; Wang, G.; Zhou, J.; Yu, J.; Ye, C.; Gan, L.; Zhang, D.; Chu, S.; Gu, L.; Shao, M.; Huang, B.; Fan, Z., Site-selective growth of fcc-2H-fcc copper on unconventional phase metal nanomaterials for highly efficient tandem $CO_2$

electroreduction. *Adv. Mater.* **2024**, *36*, 2402979.

33. Li, J.; Chen, Y.; Yao, B.; Yang, W.; Cui, X.; Liu, H.; Dai, S.; Xi, S.; Sun, Z.; Chen, W.; Qin, Y.; Wang, J.; He, Q.; Ling, C.; Wang, D.; Zhang, Z., Cascade dual sites modulate local CO coverage and hydrogen-binding strength to boost $CO_2$ electroreduction to ethylene. *J. Am. Chem. Soc.* **2024**, *146*, 5693-5701.

34. Xiong, W.; Si, D.; Li, H.; Song, X.; Wang, T.; Huang, Y.; Liu, T.; Zhang, T.; Cao, R., Steering $CO_2$ electroreduction selectivity U-turn to ethylene by Cu-Si bonded interface. *J. Am. Chem. Soc.* **2024**, *146*, 289-297.

35. Kim, J.; Lee, T.; Jung, H.; Kim, M.; Eo, J.; Kang, B.; Jung, H.; Park, J.; Bae, D.; Lee, Y.; Park, S.; Kim, W.; Back, S.; Lee, Y.; Nam, D., Vitamin C-induced $CO_2$ capture enables high-rate ethylene production in $CO_2$ electroreduction. *Nat. Commun.* **2024**, *15*, 192.

36. Li, H.; Yu, P.; Lei, R.; Yang, F.; Wen, P.; Ma, X.; Zeng, G.; Guo, J.; Toma, F.; Qiu, Y.; Geyer, S.; Wang, X.; Cheng, T.; Drisdell, W., Facet-selective deposition of ultrathin $Al_2O_3$ on copper nanocrystals for highly stable $CO_2$ electroreduction to ethylene. *Angew. Chem. Int. Ed.* **2021**, *60*, 24838-24843.

37. Chen, R.; Zu, X.; Zhu, J.; Zhao, Y.; Li, Y.; Hu, Z.; Wang, S.; Fan, M.; Zhu, S.; Zhang, H.; Ye, B.; Sun, Y.; Xie, Y., Dynamically reconstructed triple-copper-vacancy associates confined in Cu nanowires enabling high-rate and selective $CO_2$ electroreduction to $C_{2+}$ products. *Adv. Mater.* **2024**, *36*, e2314209.

38. Jeon, H.; Timoshenko, J.; Rettenmaier, C.; Herzog, A.; Yoon, A.; Chee, S.; Oener, S.; Hejral, U.; Haase, F.; Cuenya, B., Selectivity control of Cu nanocrystals in a gas-fed flow cell through $CO_2$ pulsed electroreduction. *J. Am. Chem. Soc.* **2021**, *143*, 7578-7587.

39. Hong, S.; Abbas, H.; Jang, K.; Patra, K.; Kim, B.; Choi, B.; Song, H.; Lee, K. B.; Choi, P.; Ringe, S., Tuning the $C_1/C_2$ selectivity of electrochemical $CO_2$ reduction on Cu–$CeO_2$ nanorods by oxidation state control. *Adv. Mater.* **2023**, *35*, 2208996.

40. Liu, C.; Zhang, M.; Li, J.; Xue, W.; Zheng, T.; Xia, C.; Zeng, J., Nanoconfinement

engineering over hollow multi-shell structured copper towards efficient electrocatalytical C-C coupling. *Angew. Chem. Int. Ed.* **2022**, *61*, e202113498.

41. Cai, R.; Sun, M.; Yang, F.; Gu, D.; Ju, M.; Chen, Y.; Gu, M.; Huang, B.; Yang, S., Engineering Cu(I)/Cu(0) interfaces for efficient ethanol production from $CO_2$ electroreduction. *Chem* **2024**, *10*, 211-233.

42. Heim, G.; Bruening, M.; Musgrave, C.; Goddard, W.; Peters, J.; Agapie, T., Potassium ion modulation of the Cu electrode-electrolyte interface with ionomers enhances $CO_2$ reduction to $C_{2+}$ products. *Joule* **2024**, *8*, 1312-1321.

43. Zang, H.; Zhao, Y.; Liu, C.; Lu, H.; Yu, N.; Geng, B., Hierarchical cavity Cu nanostructures with coordinative microenvironment engineering for pH-universal electrocatalytic CO-to-$C_{2+}$ conversion. *Adv. Funct. Mater.* **2025**, *35*, 2504400.

44. Hou, T.; Zhu, J.; Gu, H.; Li, X.; Sun, Y.; Hua, Z.; Shao, R.; Chen, C.; Hu, B.; Mai, L.; Chen, S.; Wang, D.; Zhang, J., Switching $CO_2$ electroreduction toward $C_{2+}$ products and $CH_4$ by regulating the dimerization and protonation in platinum/copper catalysts. *Angew. Chem. Int. Edit.* **2025**, *64*, e202424749.

45. Zhao, Z.; Zhang, Y.; Li, J.; Yao, B.; Zhang, H.; Zhang, Z., Enhanced active hydrogen absorption and stabilized Cu(I) species over Cu-O-Ce bridges boosting electrocatalytic $CO_2$ reduction to ethylene. *Angew. Chem. Int. Edit.* **2025**, *64*, e202510383.
